# Dietary diversity and the triple burden of malnutrition among children aged 6–23 months in 62 low- and middle-income countries: an analysis of Demographic and Health Surveys

**DOI:** 10.7189/jogh.16.04197

**Published:** 2026-07-24

**Authors:** Xinli Song, Qian Chen, Zhiying Song, Qing Han, Yi Zhang, Ruolin Wang, Jianuo Jiang, Li Chen, Jieyu Liu, Jun Ma, Yanhui Dong

**Affiliations:** 1 Institute of Child and Adolescent Health, School of Public Health, Peking University, National Health Commission Key Laboratory of Reproductive Health, Beijing, China; 2 Yili Kazakh Autonomous Prefecture Center for Disease Control and Prevention, Yili, Xinjiang, China; 3 Department of Pediatric Cardiology, Beijing Anzhen Hospital, Capital Medical University, Beijing, China; 4 Institute of Population Research, Peking University, Beijing, China; 5 Department of Social Policy and Intervention, University of Oxford, Oxford, UK

**Keywords:** minimum dietary diversity, infant and young child feeding, stunting, underweight, low- and middle-income countries

## Abstract

**Background:**

The 2021 World Health Organization and United Nations Children’s Fund definition of minimum dietary diversity (MDD) includes breast milk as an eighth food group. However, cross-country evidence under this revised definition remains limited. We estimated MDD achievement among children in low- and middle-income countries (LMICs) and examined its associations with anaemia, stunting, underweight, and overweight/obesity (OWOB).

**Methods:**

We analysed the most recent Demographic and Health Survey completed between 2000 and 2023 in 62 LMICs, comprising 123,569 children aged 6–23 months. We constructed MDD from standard DHS recode food-consumption variables according to the 2021 WHO/UNICEF definition, with achievement defined as consumption of at least five of eight food groups in the previous 24 hours. We estimated associations using survey-weighted logistic regression with country fixed effects and prespecified covariates. Complementary analyses included restricted cubic splines, a two-stage individual-participant-data meta-analysis, and ecological analyses.

**Results:**

Overall, 27.0% of children achieved MDD and 73.0% did not, with rates increasing from 17.7% in low-income countries to 42.9% in upper-middle-income countries. Binary MDD achievement was associated with lower odds of stunting across income groups (adjusted odds ratio (aOR) ranging from 0.677 to 0.822) and underweight (aOR ranging from 0.695 to 0.782). Continuous-score and categorical analyses showed similar patterns, whereas individual-level with anaemia and OWOB were not consistent. National MDD prevalence was inversely correlated with stunting (weighted *R* = −0.686; *P* < 0.001), underweight (weighted *R* = −0.678; *P* < 0.001), and anaemia (weighted *R* = −0.373; *P* = 0.003), but not with OWOB (weighted *R* = 0.068; *P* = 0.597).

**Conclusions:**

In our study, MDD achievement was low across 62 LMICs and varied substantially across countries and by urban or rural residence and household wealth. Greater dietary diversity was associated with lower odds of stunting and underweight across the primary income-stratified models, whereas individual-level associations with anaemia and OWOB were not consistently observed across model specifications. Policies should prioritise equitable access to affordable, nutrient-dense complementary foods, particularly in sub-Saharan Africa and socioeconomically disadvantaged populations.

Addressing malnutrition among children under five years of age is a global public health and development priority [1]. The period from 6 to 23 months is critical for complementary feeding and child nutrition, as growth faltering may begin while rapid development continues [2]. Global analyses point to a convergence of overnutrition, undernutrition, and micronutrient deficiencies within the same populations, often termed the triple burden of malnutrition [3]. This challenge is particularly acute in low- and middle-income countries (LMICs). In South Asia, recent estimates indicate that about 30.5% of children under five are stunted and 26% are underweight [4]. Simultaneously, the prevalence of overweight/obesity (OWOB) among children under five years of age across LMICs averages approximately 8.6%, demonstrating the simultaneous presence of under- and overnutrition [5]. Anaemia affected roughly 24% of the global population in 2021, with the highest burdens in sub-Saharan Africa and South Asia, and among preschool-aged children [6].

Diet is a key modifiable determinant of child nutrition. The World Health Organization (WHO) operationalises dietary diversity for children aged 6–23 months through the minimum dietary diversity (MDD) indicator, measured as the proportion who consumed foods from at least five of eight recommended food groups in the previous 24 hours [7]. In 2021, WHO and the United Nations Children’s Fund revised the indicator by adding breast milk as an eighth food group and changing the threshold from four of seven to five of eight groups [8]. The concept of MDD serves core programmatic functions, including tracking micronutrient adequacy, monitoring interventions, and targeting support to populations in need [9]. Diets meeting MDD remain uncommon worldwide, particularly in LMICs; for example, only about one quarter of children meet MDD in parts of South Asia, and roughly one fifth do so in some West African settings [10,11]. Marked regional and socioeconomic disparities persist, with well-documented gradients by maternal education, household wealth, maternal employment, and media exposure [12–14]. However, most large cross-country assessments of MDD in LMICs have relied on the 2008 definition (≥4 of 7 food groups) [15,16], and nationally representative estimates using the 2021 revision (≥5 of 8 groups, including breast milk) remain scarce.

Evidence based on the 2008 definition of MDD suggests that greater dietary diversity is associated with lower risks of stunting, underweight, and anaemia [17–19], largely through improved micronutrient density and the inclusion of animal-source foods. Over the past decade, however, LMICs have accounted for most of the global rise in child OWOB, a trend largely driven by transformations in the food system and the widespread marketing and availability of energy-dense, ultra-processed foods [20]. Importantly, the MDD indicator emphasises the number of food groups consumed, while remaining insensitive to the quality of foods within those groups [21]. Yet, previous studies on MDD have focused almost exclusively on undernutrition-related outcomes [17–19], with little attention to its potential relationship with overnutrition. Given the triple burden of malnutrition faced by many LMICs, it is critical to examine the relationship between MDD and child nutrition outcomes, which may be essential not only to refine the interpretive validity and policy relevance of MDD, but also to inform interventions that address the triple burden of malnutrition in an integrated manner.

To address this knowledge gap, we retrieved nationally representative survey data from 62 LMICs collected between 2000 and 2023 to estimate MDD achievement using the 2021 WHO and UNICEF definition, describe cross-country variation, and quantify inequalities by income group, household wealth, sex, and residence, while comparing results with the 2008 definition. We also sought to compare food-group consumption profiles and their relationships with MDD achievement across income strata and to evaluate associations between MDD and anaemia, stunting, underweight, and OWOB at individual and national levels.

## METHODS

We conducted a cross-sectional secondary analysis of public-use data from the Demographic and Health Surveys (DHS) Program, focusing on the individual child record files. We reported the study in accordance with the STROBE statement (Checklist S1 in the **Online Supplementary Document**) [22]. The DHSs are standardised, nationally representative household surveys that collect data on population, health, and nutrition across LMICs. They employ a two-stage stratified cluster sampling design: first, strata are defined by region and urban/rural residence; second, primary sampling units are selected with probability proportional to size, followed by the random selection of approximately 25 households per unit.

To use the most recent available survey for each country, we screened 114 surveys from 74 countries and retained only the most recent survey for each country conducted between 2000 and 2023. We excluded six countries with surveys predating 2000 (Thailand, Paraguay, Central African Republic, Brazil, Uzbekistan, and Kazakhstan) and six lacking dietary data (Colombia, Morocco, Nicaragua, Türkiye, Cambodia, and Moldova), resulting in a final analytic data set of 62 countries. According to the World Bank fiscal year 2024 classification based on 2022 Atlas GNI *per capita*, these included 18 low-income countries (LICs), 33 lower-middle-income countries (L-MICs), and 11 upper-middle-income countries (U-MICs), with most surveys conducted after 2010 (5 in 2000–2009, 39 in 2010–2019, and 18 in 2020–2023).

The final analytic sample comprised 123,569 children aged 6–23 months from 62 countries. Analytic denominators varied across descriptive and association analyses according to the availability of the exposure, outcome, and prespecified covariates. Outcome availability likewise differed across countries and children. Anthropometric variables were unavailable for Indonesia and the Philippines, while haemoglobin measurements were unavailable in 11 countries (Bangladesh, Chad, Comoros, Dominica, Ethiopia, Indonesia, Kenya, Pakistan, Papua New Guinea, the Philippines, and Senegal). Following outcome-specific complete-case exclusions, the effective sample sizes were 85,602 for OWOB, 85,788 for stunting, 86,685 for underweight, and 65,853 for anaemia (**Online Supplementary Document**).

### Study variables

#### Child malnutrition definition

We selected the outcome variables to reflect the triple burden of malnutrition among children aged 6–23 months, encompassing indicators of undernutrition (stunting and underweight), overnutrition (OWOB), and micronutrient deficiency (anaemia). We used DHS-supplied height-for-age (HAZ), weight-for-age (WAZ), and weight-for-height (WHZ) z-scores, standardised according to the WHO Child Growth Standards [23]. We treated values outside the WHO flag limits (HAZ <−6 or >6, WAZ <−6 or >5, and WHZ <−5 or >5) as missing.

Using these DHS-supplied variables, we classified stunting as HAZ < −2, underweight as WAZ < −2, and OWOB as WHZ > 2, encompassing overweight (2 < WHZ ≤ 3) and obesity (WHZ > 3). The DHS recode files also supplied the haemoglobin-based anaemia category; we defined any anaemia by combining the mild, moderate, and severe DHS categories (haemoglobin <110 g/L). We coded all outcomes dichotomously and excluded children with missing or implausible underlying measurements from the corresponding analysis.

#### Minimum dietary diversity definition

We constructed the primary MDD indicator from standard DHS recode food-consumption variables in accordance with the 2021 WHO/UNICEF guideline for assessing infant and young child feeding practices [8]. A defining feature of this updated framework is the formal inclusion of breast milk as the first food group, thereby expanding the evaluation system to eight food groups. Consequently, MDD attainment was defined as the consumption of foods from at least 5 of the 8 groups during the preceding 24 hours [24–26]. To ensure rigorous consistency across the 62 countries, we relied exclusively on standardised variables from the DHS Standard Recode files (Text S1 in the **Online Supplementary Document**) and adhered to the WHO principle of ‘any consumption’, imposing no minimum portion-size threshold. The specific food groups comprised: (1) breast milk; (2) grains, roots, and tubers; (3) pulses, nuts, and seeds; (4) dairy products (excluding breast milk); (5) flesh foods; (6) eggs; (7) vitamin A–rich fruits and vegetables; and (8) other fruits and vegetables. In contrast, the legacy MDD indicator based on the 2008 WHO/UNICEF guidelines excludes breast milk as a standalone component, comprising only seven food groups; accordingly, the attainment threshold under this definition was set at the consumption of at least 4 of the 7 groups.

#### Explanatory variables

Guided by UNICEF's Conceptual Framework on the Determinants of Maternal and Child Nutrition [27], we pre-specified covariates a priori to represent enabling, underlying, and child-level determinants that may confound associations with child dietary outcomes and to avoid data-driven screening. Covariate selection was further informed by prior literature [28–31]. Covariates comprised household wealth, maternal education, maternal age at first birth, urban or rural residence, piped water, improved sanitation, clean cooking fuel, child age group, sex, birth order, perceived size at birth, and fever in the preceding two weeks. The same fixed set was applied in all models, including income-stratified analyses, to preserve comparability across strata.

### Statistical analyses

All statistical analyses were conducted in *R*, version 4.4.2 (R Core Team, Vienna, Austria) using the survey package, explicitly accounting for the multistage, stratified cluster sampling design of the DHS. For descriptive analyses, survey weights were applied to generate weighted population characteristics. We computed frequencies and weighted percentages for all sociodemographic and health-related covariates, and constructed bivariate contingency tables by MDD status (achievers *vs* non-achievers). Column percentages within MDD groups were used to characterise differences in covariate distributions, and group differences were evaluated using the Rao–Scott χ^2^ test (a design-adjusted version of the Pearson χ^2^ test) to account for clustering and non-independence in complex survey data.

We constructed complex survey design objects using primary sampling unit, stratum, and sampling-weight variables. For strata containing a single primary sampling unit, we used the ‘survey’ package's *survey.lonely.psu = “adjust”* option, which centres the stratum at the overall mean rather than the stratum mean for variance estimation. We calculated weighted prevalence estimates and 95% confidence intervals (CIs) after excluding missing values. To prevent countries with larger survey samples from dominating descriptive cross-country summaries, we calculated income-group, sex, residence, and wealth estimates as arithmetic means of country-specific survey-weighted prevalences.

To investigate the independent associations between the new MDD and the four health outcomes (OWOB, stunting, underweight, and anaemia), we constructed survey-weighted logistic regression models stratified by World Bank income groups: LICs, L-MICs, and U-MICs. Accordingly, all models adjusted for child biological and developmental characteristics (age group (6–11, 12–17, and 18–23 months), sex, birth order, and perceived size at birth), recent morbidity (fever in the past two weeks), maternal age at first birth, maternal education, household wealth index, place of residence (urban/rural), and key environmental/service indicators (use of clean cooking fuel, access to improved sanitation, and piped water), in addition to country fixed effects. The same covariate set was applied in all models, including income-stratified analyses, to preserve comparability across strata.

The MDD exposure was operationalised in three forms: binary achievement (yes or no), a continuous score, and three fixed categories: low (0–2 food groups), medium (3–5 food groups), and high (6–8 food groups). We estimated adjusted odds ratios (aORs) and 95% CIs for each outcome. Potential nonlinear dose-response relationships between the continuous MDD score and each outcome were examined using restricted cubic splines within the survey-weighted logistic regression framework. Four knots were placed at the 5th, 35th, 65th, and 95th percentiles, and the income-group-specific median (50th percentile) was used as the reference. We used mean-standardised weights and cluster-robust covariance matrices and assessed overall and nonlinear associations using design-based Wald tests.

As a supplementary sensitivity analysis, we conducted a two-stage individual participant data (IPD) meta-analysis. Design-adjusted country-specific log odds ratios and standard errors were obtained in the first stage and pooled within income groups using restricted-maximum-likelihood random-effects models in the second stage. Heterogeneity was described using Cochran's Q and I2. At the ecological level, we used the World Bank mean birth population for 2018–2022 as the analytical weight and examined associations between national MDD prevalence and outcome prevalence using weighted Pearson correlations and weighted linear regression.

To describe potential selection related to missing MDD data, we compared sex, residence, and household wealth between children with and without MDD data using design-adjusted Rao-Scott χ^2^ tests. Sensitivity analyses used the 2008 MDD definition in binary and continuous-score models, survey-weighted tertiles under both frameworks, and age-stratified analyses. Effect modification by household wealth, residence, and geographic region was evaluated by adding one interaction term at a time to continuous-score models. These diagnostic and subgroup analyses were interpreted as supplementary (Tables S3, S7, S8, S10–13, and S16 in the **Online Supplementary Document**). All tests were two-sided, with *P* < 0.05 denoting statistical significance.

All data cleaning, statistical modelling, and visualisation were conducted in R version 4.4.2 (R Core Team, Vienna, Austria) using the ‘survey’, ‘rms’, ‘meta’, ‘weights’, and ‘tidyverse’ packages.

## RESULTS

### Sample characteristics by MDD achievement status

A total of 123 569 children aged 6–23 months were included. In weighted analyses, 73.0% did not meet the MDD threshold and 27.0% met it. MDD achievement differed by child age, perceived size at birth, birth order, recent fever, maternal age at first birth, maternal education, residence, household wealth, cooking fuel, sanitation, and drinking-water source (all *P* < 0.05), but not by child sex (*P* = 0.29) (**Table 1**). Values in **Table 1** are weighted frequencies and weighted column percentages; denominators vary because of variable-specific missing data and should not be interpreted as exact unweighted participant counts.

**Table 1:** Characteristics of children aged 6–23 months by minimum dietary diversity status, presented as n (weighted %)*

	Overall	Did not meet MDD	Met MDD	*P*-value†
**Child’s age in months**				<0.001
6–11	41,767 (33.8)	33,825 (37.5)	7,942 (23.8)	
12–17	43,365 (35.1)	30,005 (33.3)	13,360 (40.1)	
18–23	38,437 (31.1)	26,409 (29.3)	12,028 (36.1)	
**Child’s sex**				0.29
Male	63,483 (51.4)	46,464 (51.5)	17,020 (51.1)	
Female	60,086 (48.6)	43,775 (48.5)	16,311 (48.9)	
**Perceived size at birth**				<0.001
Small/very small	30,521 (25.1)	22,062 (24.9)	8,459 (25.8)	
Average	69,650 (57.3)	50,639 (57.1)	19,011 (57.9)	
Large/very large	19,357 (15.9)	14,360 (16.2)	4,997 (15.2)	
Do not know	2,041 (1.7)	1,695 (1.9)	346 (1.1)	
**Birth order**				<0.001
1	33,909 (27.4)	24,099 (26.7)	9,810 (29.4)	
2	29,465 (23.8)	20,620 (22.9)	8,845 (26.5)	
3	20,794 (16.8)	14,797 (16.4)	5,997 (18.0)	
≥4	39,400 (31.9)	30,723 (34.0)	8,677 (26.0)	
**Recent fever (last two weeks)**				0.03
No	89,478 (75.4)	65,939 (75.6)	23,539 (74.9)	
Yes	29,102 (24.5)	21,241 (24.3)	7,860 (25.0)	
Do not know	96 (0.1)	80 (0.1)	15 (0.0)	
**Maternal age at first birth**				<0.001
13–19 years	57,918 (47.1)	43,950 (49.0)	13,967 (42.1)	
20–29 years	61,353 (49.9)	43,426 (48.4)	17,927 (54.0)	
30–39 years	3,554 (2.9)	2,287 (2.5)	1,268 (3.8)	
40–49 years	105 (0.1)	72 (0.1)	33 (0.1)	
**Maternal education**				<0.001
No formal education	31,664 (26.6)	26,605 (30.7)	5,059 (15.6)	
Primary	30,460 (25.6)	22,584 (26.1)	7,877 (24.3)	
Secondary	44,042 (37.0)	29,865 (34.5)	14,177 (43.7)	
Higher	12,908 (10.8)	7,616 (8.8)	5,292 (16.3)	
**Place of residence**				<0.001
Urban	42,119 (34.1)	27,354 (30.3)	14,766 (44.3)	
Rural	81,450 (65.9)	62,885 (69.7)	18,564 (55.7)	
**Household wealth index**				<0.001
Poorest	27,979 (22.6)	22,345 (24.8)	5,634 (16.9)	
Poorer	25,946 (21.0)	19,862 (22.0)	6,084 (18.3)	
Middle	25,043 (20.3)	18,375 (20.4)	6,668 (20.0)	
Richer	23,455 (19.0)	16,279 (18.0)	7,176 (21.5)	
Richest	21,146 (17.1)	13,378 (14.8)	7,768 (23.3)	
**Cooking fuel type**				<0.001
Solid/unclean fuel	79,102 (64.0)	62,102 (68.8)	17,000 (51.0)	
Clean fuel	33,471 (27.1)	20,523 (22.7)	12,948 (38.8)	
Missing/other	10,997 (8.9)	7,614 (8.4)	3,382 (10.1)	
**Sanitation facility**				<0.001
Unimproved	40,870 (33.1)	33,579 (37.2)	7,291 (21.9)	
Improved	72,046 (58.3)	49,869 (55.3)	22,177 (66.5)	
Missing/other	10,653 (8.6)	6,791 (7.5)	3,862 (11.6)	
**Drinking water source**				<0.001
Non-piped	87,063 (70.5)	67,128 (74.4)	19,935 (59.8)	
Piped	28,321 (22.9)	17,816 (19.7)	10,505 (31.5)	
Missing/other	8,185 (6.6)	5,295 (5.9)	2,890 (8.7)	

MDD – minimum dietary diversity

*Values are weighted frequencies and weighted column percentages. Denominators vary because of variable-specific missing data and should not be interpreted as exact unweighted participant counts.

†Calculated using the Rao-Scott χ^2^ test to account for the complex survey design (clustering and stratification).

Comparisons of sex, residence, and household wealth between children with and without MDD data varied across countries, with no uniform direction (Table S3 in the **Online Supplementary Document**).

### Cross-country distribution of malnutrition

Across countries with available data, prevalence ranged from 1.1% to 29.3% for OWOB, 2.8% to 51.7% for stunting, 0.2% to 41.9% for underweight, and 21.3% to 86.2% for anaemia (Table S6 in the **Online Supplementary Document**).

### MDD achievement across income groups and countries

**Figure 1:**
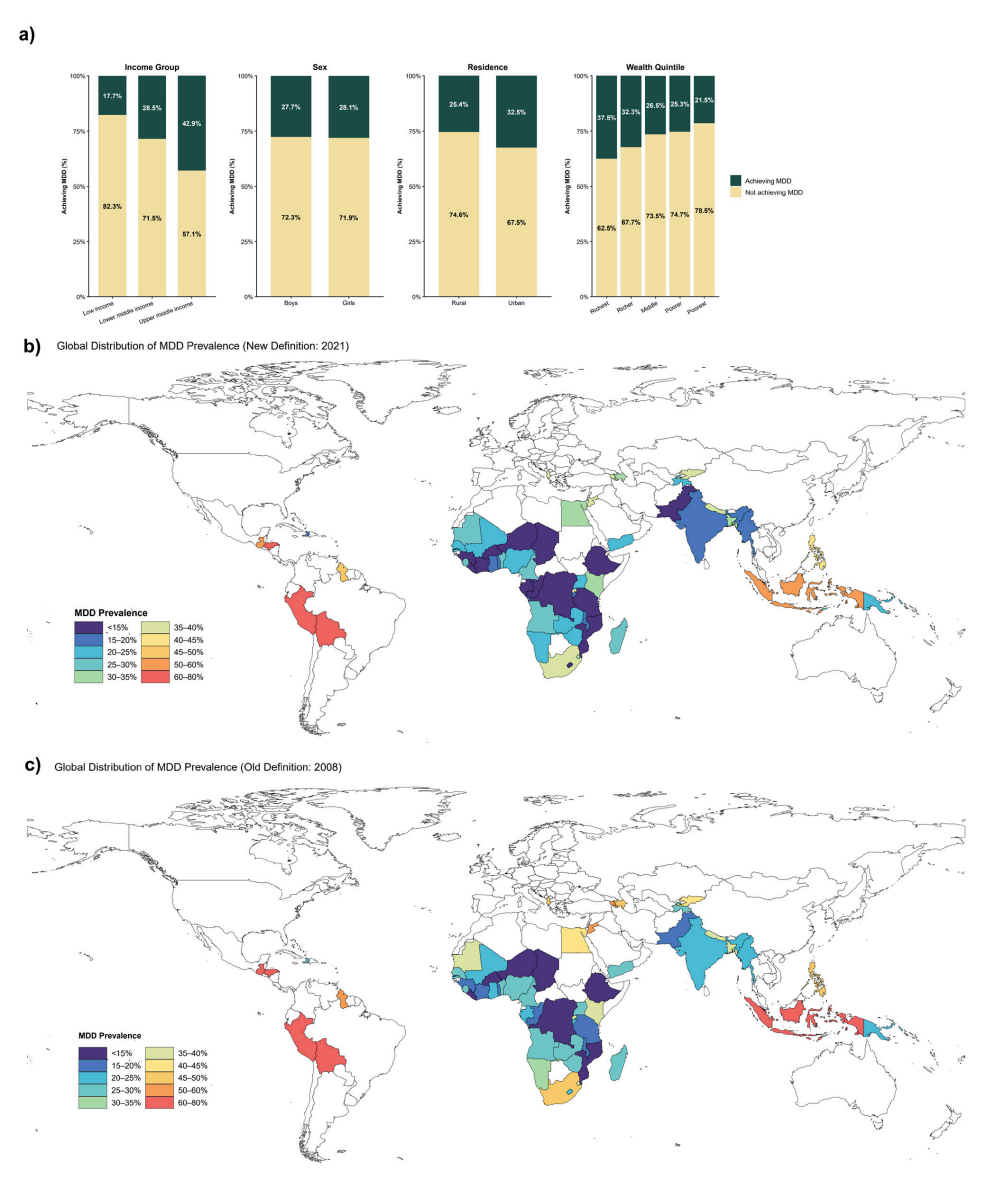
MDD achievement in 62 LMICs under the 2021 and 2008 definitions. **Panel A.** Achievement by country income group, child sex, residence, and household wealth. **Panel B.** Country-specific prevalence under the 2021 definition. **Panel C.** Country-specific prevalence under the 2008 definition. LMICs – low- and middle-income countries, MDD – minimum dietary diversity.

In country-equal descriptive summaries, MDD achievement varied substantially by subgroup and country. Prevalence increased with national income, from 17.7% in low-income countries to 42.9% in upper-middle-income countries. Rates were similar among boys and girls (27.7% *vs* 28.1%). Urban children had higher achievement than rural children (32.5% *vs* 25.4%), and achievement increased monotonically across household wealth quintiles, from 21.5% in the poorest to 37.5% in the richest quintile (**Figure 1**, Panel A).

Under the 2021 definition, country-specific MDD achievement ranged from 7.3% in Liberia to 69.2% in the Maldives. Low achievement was concentrated in parts of West and Central Africa; Burkina Faso was also among the lowest-prevalence countries (7.8%). By contrast, several countries outside Africa had markedly higher achievement, including Peru (68.0%) and Bolivia (63.9%) in South America (**Figure 1**, Panel B).

The 2008 definition (at least four of seven food groups) yielded systematically higher country-level achievement than the 2021 definition (at least five of eight food groups): the country-equal mean increased from 27.9% to 33.1%. Liberia had the lowest achievement under both definitions, while the highest-achieving country shifted from the Maldives under the 2021 definition to São Tomé and Príncipe under the 2008 definition (**Figure 1**, Panel C).

### Food-group profiles and correlates of MDD

Food-group consumption and its relationship with MDD achievement differed across World Bank income groups. In the radar chart (Figure S1, Panel A in the **Online Supplementary Document**) of eight major food groups (breast milk; grains/roots/tubers; legumes/nuts; dairy; flesh foods including meat, poultry, fish, and other animal sources; eggs; vitamin A-rich fruits/vegetables; and other fruits/vegetables), LICs had the highest reliance on breast milk (83.9%) alongside high staple consumption (grains/roots/tubers: 73.0%), but markedly lower intake of several nutrient-dense groups, especially eggs (10.1%), dairy (28.1%), and other fruits/vegetables (15.2%). U-MICs had a more diverse profile, with lower breast milk (56.8%) but substantially higher consumption of dairy (70.6%), flesh foods (50.6%), eggs (36.5%), and other fruits/vegetables (44.3%). In general, L-MICs fell in between (*e.g.* dairy 49.1%, eggs 23.0%, other fruits/vegetables 28.1%), highlighting income-graded nutrition gaps.

Point-biserial correlations (r_pb) between intake of each food group and MDD achievement (Figure S1, Panel C in the Online Supplementary Document) remained near zero for breast milk (LICs r_pb = 0.046; L-MICs r_pb = −0.002; U-MICs r_pb = 0.119), but were consistently higher for micronutrient-rich fruits/vegetables and animal-source foods. In particular, correlations were strongest for other fruits/vegetables, vitamin A-rich fruits/vegetables, and eggs, at 0.50, 0.39, and 0.42 in LICs, 0.54, 0.48, and 0.52 in L-MICs, and 0.52, 0.54, and 0.50 in U-MICs, respectively, alongside persistently high correlations for flesh foods (0.44, 0.49, and 0.48, respectively). The heat map (Figure S1, Panel B in the **Online Supplementary Document**) similarly indicated that LICs (and, to a lesser extent, L-MICs) were dominated by breast milk and staples while several complementary groups, especially eggs and other fruits/vegetables, remained relatively infrequent. In contrast, U-MICs showed higher frequencies not only for staples but also for dairy and other non-staple groups, underscoring distinct dietary patterns by income level.

### Associations between MDD and malnutrition outcomes

**Figure 2:**
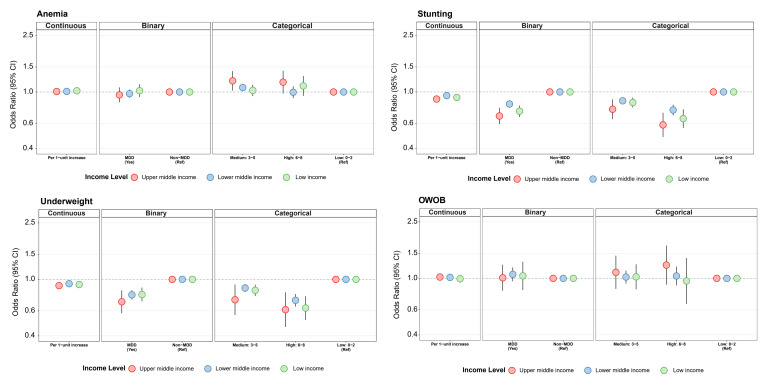
Associations of MDD with anaemia, stunting, underweight, and overweight/obesity across country income groups. MDD was modelled as a continuous score, binary achievement, and fixed low, medium, and high categories. Models incorporated survey weights and country fixed effects and were adjusted for child age, sex, birth order, perceived size at birth, recent fever, maternal age at first birth, maternal education, household wealth, residence, clean cooking fuel, improved sanitation, and piped water. MDD – minimum dietary diversity, OWOB – overweight/obesity.

Across the primary binary, continuous-score, and 2021 fixed-category specifications, greater dietary diversity was associated with lower odds of stunting and underweight in each income group, whereas associations with anaemia and OWOB were not consistent across the primary model specifications (**Figure 2**; Tables S7–9 in the **Online Supplementary Document**).

We fit survey-weighted logistic models with country fixed effects (**Figure 2**) to examine MDD associations across three specifications: binary achievement, continuous scores, and fixed categorical groups. In the binary analysis (Table S7 in the **Online Supplementary Document**), MDD achievement was consistently associated with reduced odds of stunting and underweight across all income classifications, while associations with OWOB and anaemia were not statistically significant in any income group. Specifically, in low-income countries (LICs), meeting the MDD threshold was associated with lower odds of stunting (aOR = 0.731; 95% CI = 0.666–0.802) and underweight (aOR = 0.782; 95% CI = 0.700–0.875); similar inverse associations were observed in lower-middle-income countries (L-MICs) for stunting (aOR = 0.822; 95% CI = 0.773–0.874) and underweight (aOR = 0.778; 95% CI = 0.723–0.837), and in upper-middle-income countries (U-MICs), where odds were lower for stunting (aOR = 0.677; 95% CI = 0.593–0.774) and underweight (aOR = 0.695; 95% CI = 0.577–0.837).

When MDD was modelled as a continuous score (Table S8 in the **Online Supplementary Document**), each 1-point increase was associated with lower undernutrition odds and was not associated with OWOB or anaemia outcomes. In LICs, a single-point increase in dietary diversity was associated with lower odds of stunting (aOR = 0.914; 95% CI = 0.892–0.937) and underweight (aOR = 0.920; 95% CI = 0.895–0.947); this inverse association persisted in L-MICs (stunting aOR = 0.942; underweight aOR = 0.932) and U-MICs (stunting aOR = 0.891; underweight aOR = 0.903), with all associations significant at *P* < 0.001. Conversely, continuous scores showed no statistical association with OWOB (*e.g.* LICs aOR = 0.994; *P* = 0.849) or anaemia (*e.g.* LICs aOR = 1.020; *P* = 0.157) in any income group.

Furthermore, the categorical analysis (Table S9 in the **Online Supplementary Document**) revealed that higher diversity levels (Medium: 3–5 groups; High: 6–8 groups) were associated with progressively lower odds of undernutrition compared to the low-diversity reference (0–2 groups). In LICs, the odds of stunting were lower in the medium group (aOR = 0.735; 95% CI = 0.683–0.792) and still lower in the high group (aOR = 0.526; 95% CI = 0.428–0.648), with a similar trend observed for underweight (medium aOR = 0.751; high aOR = 0.518). This pattern of stronger associations at higher diversity levels was evident across economic settings, with the high-diversity group in U-MICs showing substantially lower odds of stunting (aOR = 0.495; 95% CI = 0.402–0.610) and underweight (aOR = 0.549; 95% CI = 0.413–0.730), while no significant differences were found for OWOB or anaemia across these categories.

### Sensitivity and effect-modification analyses

Sensitivity analyses supported the primary findings without changing their interpretation (Tables S7, S8, S10, and S11–13 in the **Online Supplementary Document**). Under the legacy 2008 definition, binary and continuous-score models showed broadly similar inverse associations with stunting and underweight, whereas anaemia and OWOB showed no consistent pattern across specifications. Analyses using survey-weighted tertiles under both MDD frameworks produced similar overall patterns. Age-stratified analyses also showed broadly comparable directions, although the precision of individual estimates varied across age and income strata.

Exploratory effect-modification analyses showed heterogeneous subgroup estimates (Table S16 in the **Online Supplementary Document**). Interactions were observed for stunting by wealth, residence, and geographic region; for underweight by region; and for anaemia by region. Some stratum-specific estimates differed in direction from the primary income-stratified associations, including the lowest wealth quintile for stunting and the European subgroup for underweight. These subgroup results were considered hypothesis-generating and did not modify the interpretation of the prespecified primary models.

### Nonlinear dose-response associations

Restricted cubic spline analyses indicated several income- and outcome-specific departures from linearity rather than a uniform dose-response pattern (Figure S2 in the **Online Supplementary Document**). Signals for anaemia in L-MICs and U-MICs and for OWOB in L-MICs were not evident in the primary binary or continuous-score models, whereas spline patterns for stunting and underweight were broadly consistent with the primary inverse associations.

### Two-stage IPD meta-analysis

The two-stage IPD meta-analysis was treated as a supplementary sensitivity analysis (Tables S14 and S15 in the **Online Supplementary Document**). Estimates for the other outcomes were less consistent, and sparse data or separation produced highly imprecise country-specific and pooled estimates, particularly for underweight and OWOB.

### Ecological analysis

**Figure 3:**
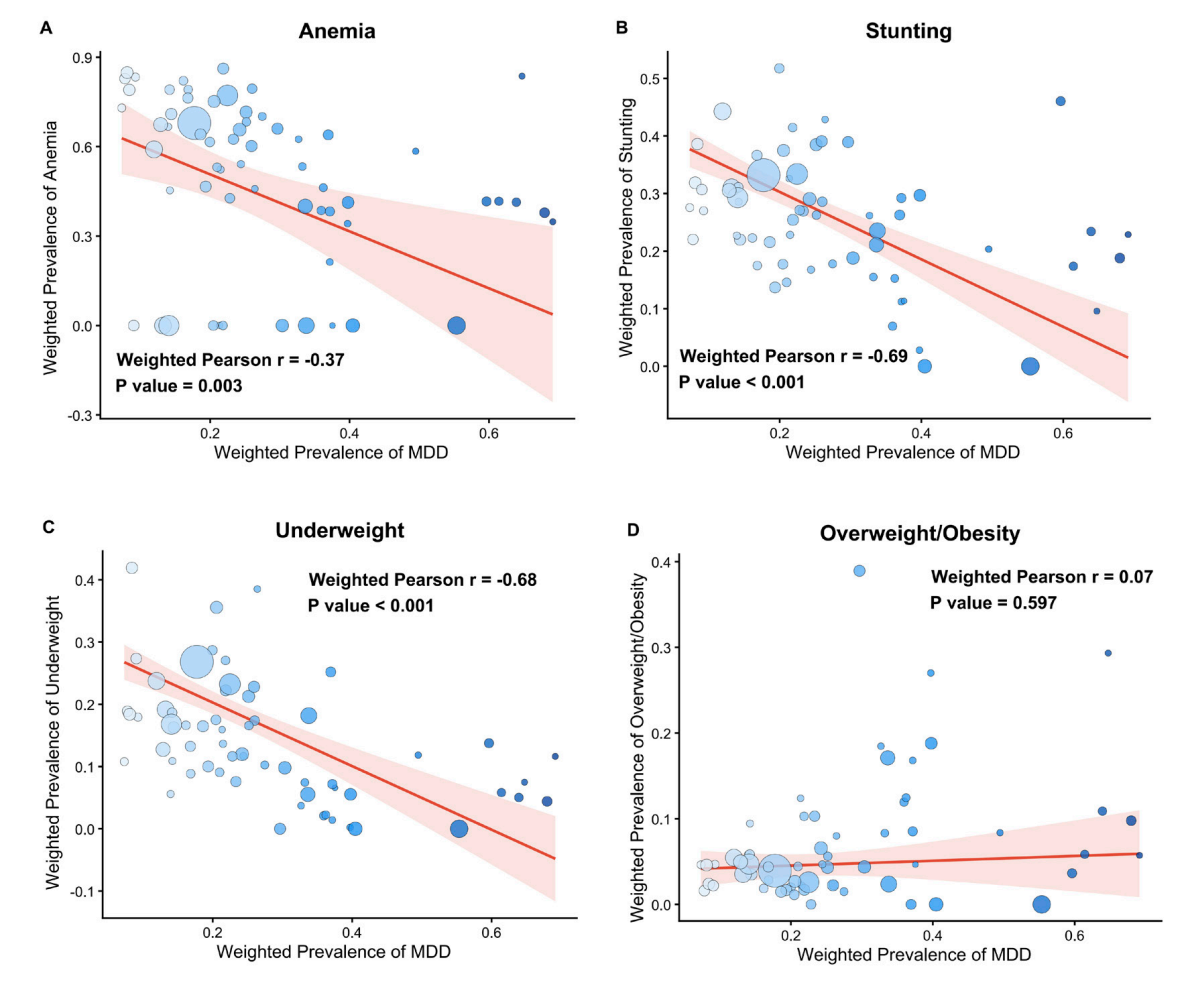
Ecological correlations between national MDD achievement and malnutrition outcomes. Birth-population-weighted scatterplots show national MDD achievement in relation to the prevalence of anaemia (**Panel A**), stunting (**Panel B**), underweight (**Panel C**), and overweight/obesity (**Panel D**). Marker size is proportional to the national birth population. MDD – minimum dietary diversity, OWOB – overweight/obesity.

At the ecological level, national MDD prevalence was inversely correlated with stunting (weighted R = −0.686; *P* < 0.001), underweight (weighted R = −0.678; *P* < 0.001), and anaemia (weighted R = −0.373; *P* = 0.003), but not with OWOB (weighted R = 0.068; *P* = 0.597) (**Figure 3**). Birth-population-weighted bubble plots showed that populous countries with lower dietary diversity contributed substantially to the observed national patterns.

## DISCUSSION

To our knowledge, this is the first study to apply the 2021 WHO/UNICEF MDD indicator to produce comparable estimates across 62 LMICs. We found that MDD achievement was lower under the 2021 definition than under the 2008 definition and varied substantially across countries and by residence and household wealth. Across the prespecified primary survey-weighted models with country fixed effects, greater dietary diversity was associated with lower odds of stunting and underweight. The stunting association was also supported by supplementary meta-analytic and ecological analyses, while estimates for other outcomes were less consistent or imprecise. Individual-level associations with anaemia and OWOB were not consistently observed across model specifications. These findings support improving access to nutrient-dense complementary foods, while avoiding causal or micronutrient-specific interpretations that cannot be established from cross-sectional data.

The country-level distributions showed substantial heterogeneity in the burden of anaemia, stunting, underweight, and OWOB (Table S6 in the **Online Supplementary Document**). Undernutrition remained prominent in many low-resource settings, whereas overnutrition was more prominent in a smaller group of countries undergoing economic and food-system change [32–39]. These descriptive patterns reflect country-level distributions and should not be interpreted as individual-level causal associations.

Only 27.0% of children met the 2021 MDD threshold during the previous 24 hours. Country-specific achievement ranged from 7.3% in Liberia to 69.2% in the Maldives. Low values were concentrated in parts of West and Central Africa, while the highest values occurred outside Africa. This heterogeneity likely reflects differences in household resources, food environments, cultural feeding practices, and survey timing [12,40,41].

Dietary pattern analyses help explain these gaps. Children in lower-income settings relied more on breast milk, while those in middle-income contexts more often accessed legumes, dairy, and meat. Supporting continued and appropriate breastfeeding remains essential in resource-constrained settings, while in some middle-income contexts changing complementary-feeding practices and broader nutrition transitions may expose children, particularly in wealthier households, to a wider range of foods [16,42,43]. Other fruits and vegetables, vitamin A-rich fruits and vegetables, and eggs showed some of the largest point-biserial correlations with MDD achievement (r_pb range across income groups: 0.39–0.54). Because these food groups are components of MDD, these correlations should not be interpreted as independent associations with nutritional outcomes. Previous evidence indicates that insufficient intake of protein-rich foods (meat and eggs) is associated with stunting and wasting [44,45] and that low consumption of vitamin A- and iron-rich foods is associated with anaemia and micronutrient deficiencies [45–47]. Biologically, these foods provide high-quality protein, vitamin B12, vitamin D, zinc, and other micronutrients essential for linear growth.

Across the primary income-stratified analyses, MDD achievement and higher MDD scores generally showed inverse associations with stunting and underweight, whereas associations with anaemia and OWOB were inconsistent. The 2021 fixed-category models also showed graded inverse associations with stunting and underweight; in LICs, for example, the odds of stunting were lower in both the medium-MDD group (aOR = 0.735; 95% CI = 0.683–0.792) and the high-MDD group (aOR = 0.526; 95% CI = 0.428–0.648) than in the low-MDD group. Greater dietary diversity may improve the availability of energy, high-quality protein, and growth-relevant micronutrients, but the cross-sectional design precludes causal interpretation [44–54]. Environmental enteric exposures, infection, portion size, and food quality within each group may also modify these associations [55–59].

The evidence for OWOB was inconsistent overall. Neither binary MDD achievement nor the continuous MDD score was associated with OWOB in the primary models, and national MDD prevalence was not correlated with national OWOB prevalence. An isolated nonlinear association in L-MICs should, therefore, be viewed as exploratory, rather than as evidence that greater dietary diversity generally increases OWOB. Likewise, the inverse country-level correlation between MDD and anaemia was not observed in the individual-level models. This difference may reflect contextual confounding and aggregation at the national level and should not be interpreted as evidence of an individual protective effect against anaemia.

Taken together, these results position improved dietary diversity as one component of strategies to address stunting and underweight, rather than as demonstrated evidence of reduced micronutrient deficiency or OWOB risk. Policies should expand equitable access to affordable, nutrient-dense complementary foods, including eggs, other animal-source foods, and vitamin A-rich fruits and vegetables, alongside support for continued breastfeeding and age-appropriate feeding. Because dietary diversity is shaped by household resources and food systems, these measures should be integrated with social protection, public health, education, and resilient food-supply policies and adapted to local cultural and economic contexts [60].

### Strengths and limitations

The main strengths of this study were its use of nationally representative surveys from 62 countries, standardised definitions, explicit accounting for complex survey designs, and triangulation across several prespecified exposure specifications and supplementary analyses. Several limitations should be considered. First, reliance on secondary data restricted the analysis to variables available in the DHS. Second, the cross-sectional design precluded causal inference and could not rule out reverse causality. Third, the 24-hour dietary recall captured only a short-term snapshot and may have been affected by season, fasting, or festive diets. Fourth, missingness diagnostics did not indicate a uniform direction of selection, but some comparisons were significant or not estimable; residual selection bias therefore cannot be excluded. Finally, some country-specific underweight and OWOB estimates in the IPD sensitivity analysis were unstable because of sparse outcomes or separation, and the corresponding pooled estimates were treated as exploratory rather than confirmatory.

## CONCLUSIONS

Among 123,569 children aged 6–23 months in 62 LMICs, MDD achievement under the 2021 definition was low and varied substantially across countries and by residence and household wealth. Across the primary income-stratified models, greater dietary diversity was associated with lower odds of stunting and underweight, whereas individual-level associations with anaemia and OWOB were not consistently observed across model specifications. Improving access to affordable, nutrient-dense complementary foods may help reduce growth faltering, particularly in sub-Saharan Africa and socioeconomically disadvantaged populations. Longitudinal research is needed to clarify causality and the quality of foods consumed within MDD food groups.

## Additional material

Online Supplementary Document

## Data Availability

**Data availability:** The de-identified data sets used in this study are available from the Demographic and Health Surveys Program after registration and approval of a research request (https://dhsprogram.com).

## References

[1] Black RE Victora CG Walker SP Bhutta ZA Christian P de Onis M Maternal and child undernutrition and overweight in low-income and middle-income countries. Lancet. 2013;382:427–51. 10.1016/S0140-6736(13)60937-X 23746772

[2] Panjwani A Heidkamp R Complementary feeding interventions have a small but significant impact on linear and ponderal growth of children in low- and middle-income countries: a systematic review and meta-analysis. J Nutr. 2017;147:2169S–78S. 10.3945/jn.116.243857 28904113

[3] The triple burden of malnutrition. Nat Food. 2023;4:925. 10.1038/s43016-023-00886-8 37985699

[4] Guo W Zgambo M Chen S Shimpuku Y Prevalence and trends of coexisting forms of malnutrition and its associated factors among children aged 6–59 months in South and Southeast Asia, 1996–2022: a cross-sectional time series study. BMC Public Health. 2025;25:2296. 10.1186/s12889-025-23482-w 40610994 PMC12224721

[5] Talukdar R Biswas S Seelamantula A Pahari S Ghosh D Sagiraju HKR Prevalence of overweight/obesity among under-five children in lower middle-income countries and assessment of the reported associated factors: a systematic review and meta-analysis. Pediatr Obes. 2025;20:e70055. 10.1111/ijpo.70055 40947305

[6] GBD 2021 Anaemia Collaborators Prevalence, years lived with disability, and trends in anaemia burden by severity and cause, 1990–2021: findings from the Global Burden of Disease Study 2021. Lancet Haematol. 2023;10:e713–34. 10.1016/S2352-3026(23)00160-6 37536353 PMC10465717

[7] World Health Organization. Child feeding: minimum dietary diversity, 6–23 months. Available: https://www.who.int/data/gho/data/indicators/indicator-details/GHO/minimum-dietary-diversity-6-23-months. Accessed: 11 July 2026.

[8] World Health Organization, United Nations Children’s Fund. Indicators for assessing infant and young child feeding practices: definitions and measurement methods. Geneva, Switzerland: World Health Organization; 2021. Available: https://www.who.int/publications/i/item/9789240018389. Accessed: 11 July 2026.

[9] Singh A Rahut DB Sonobe T Exploring minimum dietary diversity among Cambodian children using four rounds of demographic and health survey. Sci Rep. 2024;14:14719. 10.1038/s41598-024-64714-0 38926408 PMC11208556

[10] Onyango AW Borghi E de Onis M Casanovas Mdel C Garza C Complementary feeding and attained linear growth among 6–23-month-old children. Public Health Nutr. 2014;17:1975–83. 10.1017/S1368980013002401 24050753 PMC11108726

[11] Rahman MA Kundu S Rashid HO Tohan MM Islam MA Socio-economic inequalities in and factors associated with minimum dietary diversity among children aged 6–23 months in South Asia: a decomposition analysis. BMJ Open. 2023;13:e072775. 10.1136/bmjopen-2023-072775 38128933 PMC10749007

[12] Ativor PS Salu S Optimizing minimum dietary diversity: examining appropriate complementary feeding practices and influencing factors among children aged 6–23 months in Ghana; a cross-sectional study. BMC Public Health. 2025;25:559. 10.1186/s12889-025-21681-z 39934732 PMC11817239

[13] Molla W Adem DA Tilahun R Shumye S Kabthymer RH Kebede D Dietary diversity and associated factors among children (6–23 months) in Gedeo zone, Ethiopia: cross-sectional study. Ital J Pediatr. 2021;47:233. 10.1186/s13052-021-01181-7 34895268 PMC8665621

[14] Shibeshi AH Asfaw ZG The influence of minimum dietary diversity on undernutrition among children aged 6–23 months in Ethiopia: a multilevel mixed-effect analysis based on 2019 Ethiopian Mini Demographic and Health Survey. Front Public Health. 2024;12:1436683. 10.3389/fpubh.2024.1436683 39444959 PMC11496284

[15] Baye K Kennedy G Estimates of dietary quality in infants and young children (6–23 mo): evidence from demographic and health surveys of 49 low- and middle-income countries. Nutrition. 2020;78:110875. 10.1016/j.nut.2020.110875 32653760

[16] Gatica-Domínguez G Neves PAR Barros AJD Victora CG Complementary feeding practices in 80 low- and middle-income countries: prevalence of and socioeconomic inequalities in dietary diversity, meal frequency, and dietary adequacy. J Nutr. 2021;151:1956–64. 10.1093/jn/nxab088 33847352 PMC8245881

[17] Masuke R Msuya SE Mahande JM Diarz EJ Stray-Pedersen B Jahanpour O Effect of inappropriate complementary feeding practices on the nutritional status of children aged 6–24 months in urban Moshi, northern Tanzania: cohort study. PLoS One. 2021;16:e0250562. 10.1371/journal.pone.0250562 33983950 PMC8118559

[18] Li H Moosavian SP Ghanbari N Mirlohi SH Rahimlou M Association of dietary diversity and odds of anemia in children and adolescents: a systematic review and meta-analysis of observational studies. BMC Nutr. 2025;11:83. 10.1186/s40795-025-01069-3 40264235 PMC12016368

[19] Belachew A Tewabe T Under-five anemia and its associated factors with dietary diversity, food security, stunted, and deworming in Ethiopia: systematic review and meta-analysis. Syst Rev. 2020;9:31. 10.1186/s13643-020-01289-7 32051034 PMC7017616

[20] Jebeile H Kelly AS O’Malley G Baur LA Obesity in children and adolescents: epidemiology, causes, assessment, and management. Lancet Diabetes Endocrinol. 2022;10:351–65. 10.1016/S2213-8587(22)00047-X 35248172 PMC9831747

[21] Pries AM Sharma N Upadhyay A Rehman AM Filteau S Ferguson EL Energy intake from unhealthy snack food/beverage among 12–23-month-old children in urban Nepal. Matern Child Nutr. 2019;15 Suppl 4:e12775. 10.1111/mcn.12775 31225707 PMC6617731

[22] von Elm E Altman DG Egger M Pocock SJ Gøtzsche PC Vandenbroucke JP The Strengthening the Reporting of Observational Studies in Epidemiology (STROBE) statement: guidelines for reporting observational studies. J Clin Epidemiol. 2008;61:344–9. 10.1016/j.jclinepi.2007.11.008 18313558

[23] World Health Organization. WHO child growth standards: length/height-for-age, weight-for-age, weight-for-length, weight-for-height and body mass index-for-age: methods and development. Geneva, Switzerland: World Health Organization; 2006. Available: https://www.who.int/publications/i/item/924154693X. Accessed: 11 July 2026.

[24] Arimond M Ruel MT Dietary diversity is associated with child nutritional status: evidence from 11 demographic and health surveys. J Nutr. 2004;134:2579–85. 10.1093/jn/134.10.2579 15465751

[25] Khamis AG Mwanri AW Ntwenya JE Kreppel K The influence of dietary diversity on the nutritional status of children between 6 and 23 months of age in Tanzania. BMC Pediatr. 2019;19:518. 10.1186/s12887-019-1897-5 31881999 PMC6935228

[26] Frempong RB Annim SK Dietary diversity and child malnutrition in Ghana. Heliyon. 2017;3:e00298. 10.1016/j.heliyon.2017.e00298 28503669 PMC5419825

[27] United Nations Children’s Fund. UNICEF conceptual framework on maternal and child nutrition. New York, USA: United Nations Children's Fund; 2021. Available: https://www.unicef.org/documents/conceptual-framework-nutrition. Accessed: 11 July 2026.

[28] Nshimyiryo A Hedt-Gauthier B Mutaganzwa C Kirk CM Beck K Ndayisaba A Risk factors for stunting among children under five years: a cross-sectional population-based study in Rwanda using the 2015 Demographic and Health Survey. BMC Public Health. 2019;19:175. 10.1186/s12889-019-6504-z 30744614 PMC6371425

[29] Akombi BJ Agho KE Hall JJ Wali N Renzaho AMN Merom D Stunting, wasting and underweight in sub-Saharan Africa: a systematic review. Int J Environ Res Public Health. 2017;14:863. 10.3390/ijerph14080863 28788108 PMC5580567

[30] Poda GG Hsu CY Chao JC Factors associated with malnutrition among children <5 years old in Burkina Faso: evidence from the Demographic and Health Surveys IV 2010. Int J Qual Health Care. 2017;29:901–8. 10.1093/intqhc/mzx129 29045661

[31] Talukder A Factors associated with malnutrition among under-five children: illustration using Bangladesh Demographic and Health Survey, 2014 data. Children (Basel). 2017;4:88. 10.3390/children4100088 29048355 PMC5664018

[32] World Health Organization. Anaemia in children <5 years | Anaemia in women of reproductive age, by pregnancy status. Available: https://www.who.int/data/gho/data/themes/topics/GHO/anaemia-in-children-5-years. Accessed: 11 July 2026.

[33] Scott SP Chen-Edinboro LP Caulfield LE Murray-Kolb LE The impact of anemia on child mortality: an updated review. Nutrients. 2014;6:5915–32. 10.3390/nu6125915 25533005 PMC4277007

[34] de Onis M Blössner M Borghi E Global prevalence and trends of overweight and obesity among preschool children. Am J Clin Nutr. 2010;92:1257–64. 10.3945/ajcn.2010.29786 20861173

[35] Scarpa G Berrang-Ford L Galazoula M Kakwangire P Namanya DB Tushemerirwe F Identifying predictors for minimum dietary diversity and minimum meal frequency in children aged 6–23 months in Uganda. Nutrients. 2022;14:5208. 10.3390/nu14245208 36558366 PMC9786234

[36] Sánchez-Martínez LJ Charle-Cuéllar P Gado AA Dougnon AO Sanoussi A Ousmane N Impact of a simplified treatment protocol for moderate acute malnutrition with a decentralized treatment approach in emergency settings of Niger. Front Nutr. 2023;10:1253545. 10.3389/fnut.2023.1253545 38099186 PMC10719846

[37] Valente A Silva D Neves E Almeida F Cruz JL Dias CC Acute and chronic malnutrition and their predictors in children aged 0–5 years in São Tomé: a cross-sectional, population-based study. Public Health. 2016;140:91–101. 10.1016/j.puhe.2016.07.017 27576113

[38] Ekholuenetale M Okonji OC Nzoputam CI Barrow A Inequalities in the prevalence of stunting, anemia and exclusive breastfeeding among African children. BMC Pediatr. 2022;22:333. 10.1186/s12887-022-03395-y 35681131 PMC9178835

[39] Hawkes C, Harris J, Gillespie S. Changing diets: urbanization and the nutrition transition. In: 2017 Global Food Policy Report. Washington, DC, USA: International Food Policy Research Institute; 2017. p. 34–41. Available: https://hdl.handle.net/10568/146456. Accessed: 11 July 2026.

[40] Belay DG Aragaw FM Teklu RE Fetene SM Negash WD Asmamaw DB Determinants of inadequate minimum dietary diversity intake among children aged 6–23 months in sub-Saharan Africa: pooled prevalence and multilevel analysis of Demographic and Health Survey in 33 sub-Saharan African countries. Front Nutr. 2022;9:894552. 10.3389/fnut.2022.894552 35845763 PMC9284213

[41] Dangura D Gebremedhin S Dietary diversity and associated factors among children 6–23 months of age in Gorche district, southern Ethiopia: cross-sectional study. BMC Pediatr. 2017;17:6. 10.1186/s12887-016-0764-x 28068965 PMC5223415

[42] Victora CG Joseph G Silva ICM Maia FS Vaughan JP Barros FC The inverse equity hypothesis: analyses of institutional deliveries in 286 national surveys. Am J Public Health. 2018;108:464–71. 10.2105/AJPH.2017.304277 29470118 PMC5844402

[43] Langley-Evans SC Complementary feeding: should baby be leading the way? J Hum Nutr Diet. 2022;35:247–9. 10.1111/jhn.12988 35066946 PMC9303566

[44] Headey DD Palloni G Stunting and wasting among Indian preschoolers have moderate but significant associations with the vegetarian status of their mothers. J Nutr. 2020;150:1579–89. 10.1093/jn/nxaa042 32171005 PMC7269725

[45] Mya KS Kyaw AT Tun T Feeding practices and nutritional status of children age 6–23 months in Myanmar: a secondary analysis of the 2015–16 Demographic and Health Survey. PLoS One. 2019;14:e0209044. 10.1371/journal.pone.0209044 30601848 PMC6314612

[46] Donkor WES Adu-Afarwuah S Wegmüller R Bentil H Petry N Rohner F Complementary feeding indicators in relation to micronutrient status of Ghanaian children aged 6–23 months: results from a national survey. Life (Basel). 2021;11:969. 10.3390/life11090969 34575118 PMC8468967

[47] Karlsson O Kim R Hasman A Subramanian SV Consumption of vitamin-A-rich foods and vitamin A supplementation for children under two years old in 51 low- and middle-income countries. Nutrients. 2021;14:188. 10.3390/nu14010188 35011064 PMC8747127

[48] Habtegiorgis SD Petrucka P Telayneh AT Getahun DS Getacher L Alemu S Prevalence and associated factors of anemia among adolescent girls in Ethiopia: a systematic review and meta-analysis. PLoS One. 2022;17:e0264063. 10.1371/journal.pone.0264063 35324901 PMC8947116

[49] Nti CA Dietary diversity is associated with nutrient intakes and nutritional status of children in Ghana. Asian J Med Sci. 2011;2:105–9. 10.3126/ajms.v2i2.4179

[50] Rani VR Arends D Brouwer ID Dietary diversity as an indicator of micronutrient adequacy of the diet of 5–8-year-old Indian rural children. Nutr Food Sci. 2010;40:466–76. 10.1108/00346651011076974

[51] Tareke AA Temam AJ Alem A Geto Z Assefa EM Bihonegn MD The link between child dietary diversity and child anemia: the power of colorful plates. PLOS Glob Public Health. 2025;5:e0005001. 10.1371/journal.pgph.0005001 40737242 PMC12309991

[52] Koury MJ Ponka P New insights into erythropoiesis: the roles of folate, vitamin B12, and iron. Annu Rev Nutr. 2004;24:105–31. 10.1146/annurev.nutr.24.012003.132306 15189115

[53] Wang J Chang S Zhao L Yu W Zhang J Man Q Effectiveness of community-based complementary food supplement (Yingyangbao) distribution in children aged 6–23 months in poor areas in China. PLoS One. 2017;12:e0174302. 10.1371/journal.pone.0174302 28319154 PMC5358851

[54] Aboagye RG Seidu AA Ahinkorah BO Arthur-Holmes F Cadri A Dadzie LK Dietary diversity and undernutrition in children aged 6–23 months in sub-Saharan Africa. Nutrients. 2021;13:3431. 10.3390/nu13103431 34684435 PMC8537414

[55] Sarfo JO Amoadu M Gyan TB Osman AG Kordorwu PY Adams AK Acute lower respiratory infections among children under five in sub-Saharan Africa: a scoping review of prevalence and risk factors. BMC Pediatr. 2023;23:225. 10.1186/s12887-023-04033-x 37149597 PMC10163812

[56] Sulistiyowati N Tjandrarini DH Titaley CR Que BJ Hidayangsih PS Suparmi Suboptimal child healthcare practices and the development of multiple infectious diseases in children aged 24–59 months. Front Public Health. 2024;12:1340559. 10.3389/fpubh.2024.1340559 38504680 PMC10948606

[57] Sinha P Guerrant RL The costly vicious cycle of infections and malnutrition. J Infect Dis. 2024;229:1611–3. 10.1093/infdis/jiad513 37972258 PMC11175688

[58] Kishino M Hida A Chadeka EA Inoue M Osada-Oka M Matsumoto S Association between diet quality and risk of stunting among school-aged children in a *Schistosoma mansoni* endemic area of western Kenya: a cross-sectional study. Trop Med Health. 2024;52:12. 10.1186/s41182-023-00566-0 38233936 PMC10792916

[59] Demilew YM Factors associated with mothers’ knowledge on infant and young child feeding recommendation in slum areas of Bahir Dar City, Ethiopia: cross-sectional study. BMC Res Notes. 2017;10:191. 10.1186/s13104-017-2510-3 28583203 PMC5460351

[60] Afridi F Child welfare programs and child nutrition: evidence from a mandated school meal program in India. J Dev Econ. 2010;92:152–65. 10.1016/j.jdeveco.2009.02.002

