# Supplementary material for: Dietary diversity and the triple burden of malnutrition among children aged 6–23 months in 62 low- and middle-income countries: an analysis of Demographic and Health Surveys
Source: J Glob Health. 2026 Jul 24;16:04197. doi: 10.7189/jogh.16.04197 (PMC13397094; doi:10.7189/jogh.16.04197)
Supplement: Online Supplementary Document [file jogh-16-04197-s001.pdf]

Supplement to: Song X, Chen Q, Song Z, Dong Y. Dietary diversity and the triple burden of malnutrition among children aged 6–23 months in 62 low- and middle-income countries: analysis of Demographic and Health Surveys. J Glob Health. 2026;16:04197.

Online Supplementary Document

Text S1. Supplementary methods

Figures S1-S2. Supplementary figures

Tables S1-S16. Supplementary results

Checklist S1. STROBE checklist for cross-sectional studies

Text S1. Supplementary methods

Study design and data source

We conducted a secondary analysis of publicly available, de-identified individual-level child records from the Demographic and Health Surveys (DHS) Program. The DHS are standardised, nationally representative household surveys that use a multistage stratified cluster design. Strata are generally defined by geographic region and urban or rural residence; primary sampling units are selected with probability proportional to size, followed by systematic household sampling within each selected unit.

The initial search identified 114 surveys from 74 countries. We retained the most recent survey for each country, excluded six countries whose most recent survey predated 2000, and excluded six countries with no usable dietary data. The final dataset comprised the most recent eligible survey conducted between 2000 and 2023 in 62 countries: 18 low-income, 33 lower-middle-income, and 11 upper-middle-income countries under the World Bank FY2024 classification. Table S1 lists the included surveys, and Table S2 summarises country-level missingness in MDD variables.

Study population and outcome-specific samples

The final analytic sample comprised 123 569 children aged 6–23 months. Analytic denominators varied by exposure, outcome, covariate, and table-specific availability. Anthropometric variables were unavailable for Indonesia and the Philippines, and haemoglobin measurements were unavailable in 11 countries. Following outcome-specific complete-case exclusions, the effective sample sizes were 85 602 for overweight/obesity, 85 788 for stunting, 86 685 for underweight, and 65 853 for anaemia. Country-specific descriptive estimates are presented only when the supplied source values were considered reportable.

Minimum dietary diversity

Updated 2021 indicator

We constructed minimum dietary diversity (MDD) using the 2021 WHO/UNICEF indicator definition and the 24-hour dietary recall module in the DHS standard questionnaire. MDD achievement was defined as consumption of foods from at least five of eight food groups during the preceding 24 hours. Breast milk was included as a food group. Because DHS records list-based recall rather than weighed intake, no minimum portion-size threshold was applied. A food group was counted as consumed when the caregiver reported any consumption during the recall period. Responses recorded as do not know were conservatively coded as non-consumption for score derivation.

DHS does not provide complete recipe quantities for mixed dishes. We therefore used the questionnaire's respondent-level component reporting and did not attempt post hoc ingredient allocation. To maximise comparability, construction was restricted to standardised DHS recode variables. Country-specific variables were used only when their metadata allowed unambiguous mapping to one of the eight food groups.

Mapping of food groups to DHS variables

Breast milk. V404; coded as consumed when the child was currently breastfeeding

Grains, roots, and tubers. V414E, V414F, V412A, and V412B; including cereal-based fortified baby foods and porridges

Pulses, nuts, and seeds. V414O

Dairy products. V411, V411A, V414P, and V414V; excluding breast milk

Flesh foods. V414H, V414M, and V414N; including meat, poultry, organ meats, fish, and shellfish

Eggs. V414G

Vitamin A-rich fruits and vegetables. V414I, V414J, and V414K

Other fruits and vegetables. V414L; restricted to the standardised variable for cross-country consistency

Derived MDD measures and the legacy indicator

Four measures were derived for the 2021 framework: an eight-point continuous score; binary achievement at five or more food groups; fixed categories of low (0–2), medium (3–5), and high (6–8); and survey-weighted tertiles estimated within country-income-group and outcome-specific analytic strata. For sensitivity analyses, the legacy 2008 indicator excluded breast milk as a standalone group, used a seven-point score, and defined achievement at four or more food groups. Weighted tertiles under the 2008 framework were created using the same design-based procedure.

Child health outcomes

We examined four binary outcomes representing the triple burden of malnutrition. Height-for-age, weight-for-age, and weight-for-height z-scores were derived using the WHO Child Growth Standards. Under the prespecified rule applied uniformly across surveys, absolute z-scores greater than 6 were treated as missing. Stunting was defined as height-for-age z-score below -2, underweight as weight-for-age z-score below -2, and overweight/obesity as weight-for-height z-score above 2. For comparability across the included survey years, anaemia was defined using the prespecified DHS-based threshold of haemoglobin below 110 g/L. Missing or implausible measurements were not recoded as non-cases.

Covariates

Covariates were selected a priori from the literature and from variables consistently available in DHS. Models included child age group, sex, birth order, perceived size at birth, and fever in the preceding two weeks; maternal age at first birth and educational attainment; household wealth and urban or rural residence; and indicators for clean cooking fuel, improved sanitation, and piped water. Country fixed effects were included in the primary association models. The same covariate set was retained across income-stratified models to preserve comparability.

Statistical analyses

Survey design and descriptive analyses

Analyses were conducted in R version 4.4.2 using the survey package to account for primary sampling units, strata, and sampling weights. Raw DHS weights were divided by 1 000 000, and the adjust method was used for strata containing a single primary sampling unit. Weighted frequencies and percentages described the analytic sample, and Rao-Scott chi-square tests compared characteristics by MDD achievement. For cross-country summaries by income group, sex, residence, and wealth, we calculated arithmetic means of country-specific survey-weighted prevalences so that countries with larger survey samples did not dominate the results.

Primary association models and spline analyses

Survey-weighted logistic regression models estimated associations of MDD with overweight/obesity, stunting, underweight, and anaemia within World Bank income groups. MDD was modelled as binary achievement, a continuous score, and fixed low, medium, and high categories. Adjusted odds ratios and 95% confidence intervals were reported. Potential non-linear dose-response patterns were examined using restricted cubic splines with knots at the 5th, 35th, 65th, and 95th percentiles and the income-group-specific median as the reference. Mean-standardised weights and cluster-robust covariance matrices were used, and overall and non-linear associations were assessed with design-based Wald tests.

Sensitivity, meta-analytic, and ecological analyses

Sensitivity analyses repeated the principal binary and continuous-score models using the 2008 MDD definition, re-specified MDD as weighted tertiles under both frameworks, and stratified analyses by age group (6–11, 12–17, and 18–23 months). Potential effect modification by household wealth, residence, and geographic

region was assessed by adding one interaction term at a time to the continuous-score models and applying design-based joint Wald tests. These sensitivity and subgroup analyses were considered supplementary.

As a supplementary sensitivity analysis, we conducted a two-stage individual participant data meta-analysis. Design-adjusted country-specific log odds ratios and standard errors were estimated in the first stage and pooled within income groups using restricted-maximum-likelihood random-effects models in the second stage. Heterogeneity was described using Cochran's Q and I2. Sparse outcomes and separation produced highly imprecise country-specific estimates for some underweight and OWOB analyses; all meta-analytic estimates were therefore treated as exploratory rather than confirmatory.

For ecological analyses, the mean national birth population for 2018–2022, calculated from total population and crude birth rate, was used as the analytical weight. Weighted Pearson correlations and weighted linear regression related national MDD prevalence to national outcome prevalence. Bubble plots used marker size to represent birth population.

Missing data, software, and statistical threshold

Potential selection related to missing MDD data was described by comparing sex, residence, and household wealth between children with and without MDD data using design-adjusted Rao-Scott tests. These comparisons were descriptive and were not adjusted for multiple testing; a mixture of significant, non-significant, and non-estimable results was not interpreted as evidence that missingness was random. Outcome-specific analyses used complete cases for the variables required by each model. All tests were two-sided, and $P < 0.05$ denoted statistical significance.

Figure S1. Food-group profiles and correlates of minimum dietary diversity achievement across income groups. Panel A presents a radar chart of eight food groups. Panel B presents the country-by-food-group prevalence heat map. Panel C presents point-biserial correlations between each food group and minimum dietary diversity achievement. MDD - minimum dietary diversity.

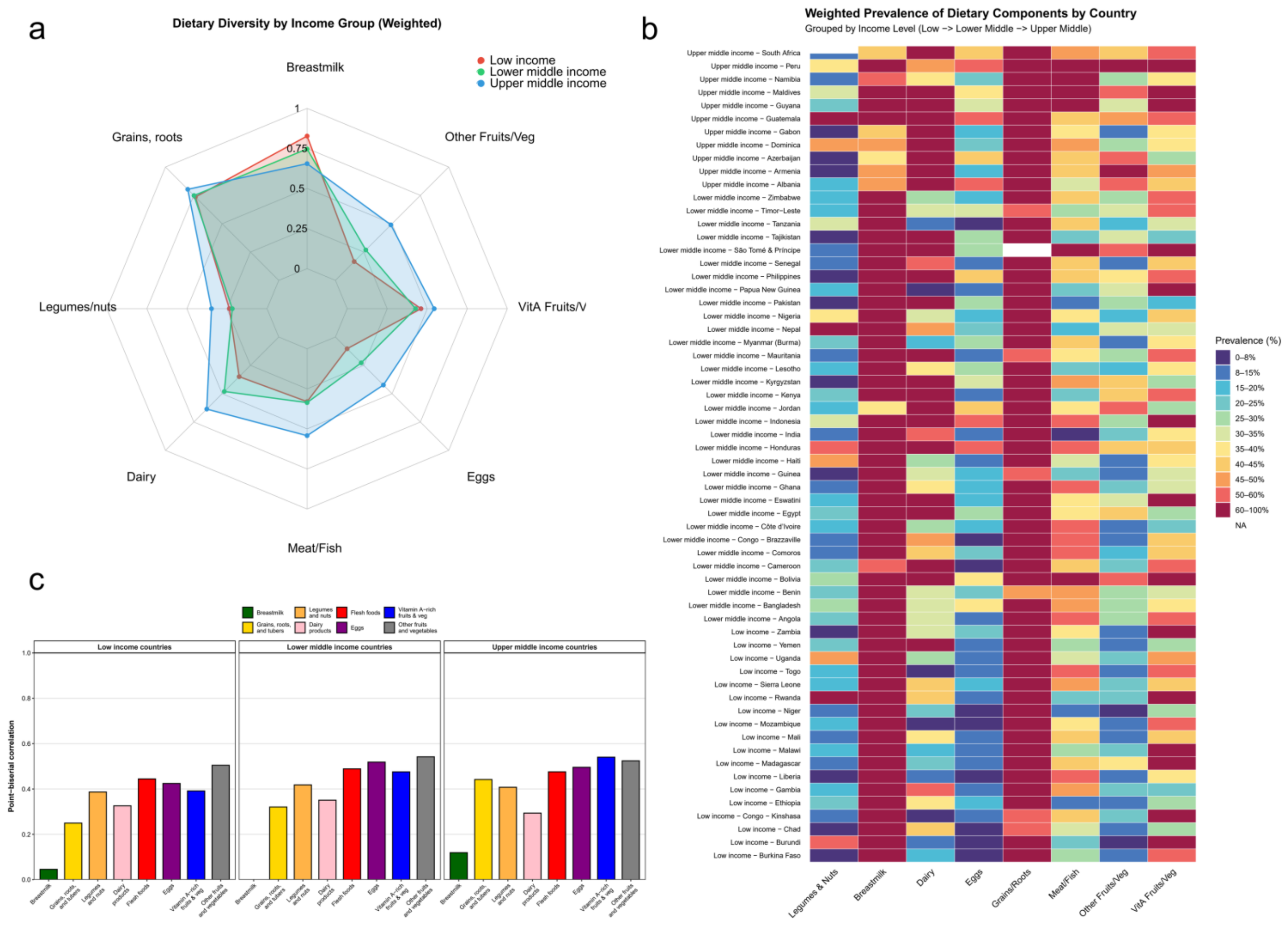

Figure S2. Restricted cubic spline curves for associations between minimum dietary diversity score and anaemia, stunting, underweight, and overweight/obesity across income groups. The reference is the income-group-specific median score. MDD - minimum dietary diversity; OWOB - overweight/obesity.

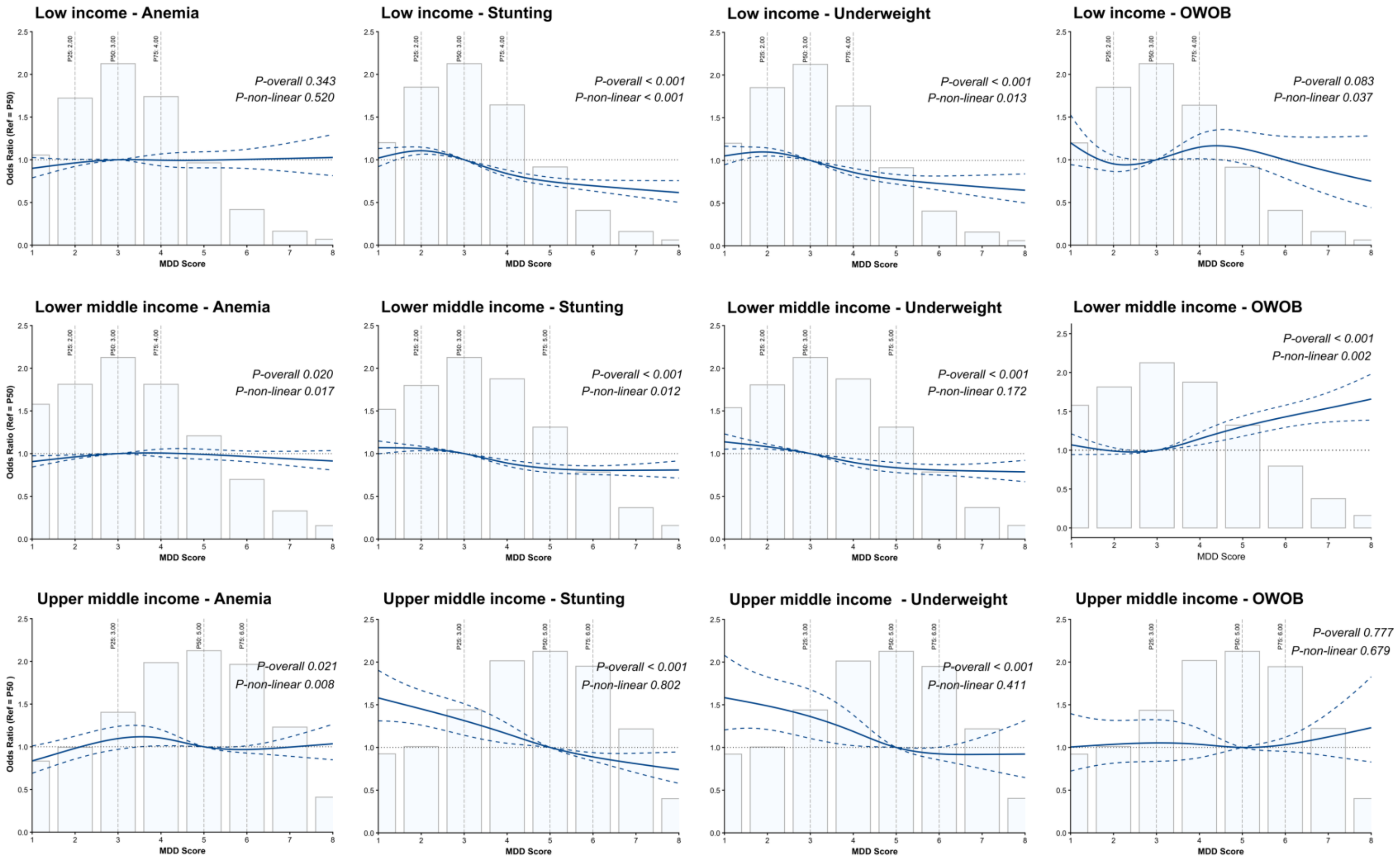

Table S1. List of 62 countries with the most recent eligible Demographic and Health Survey conducted in or after 2000.

ISO3	Survey year	DHS dataset	DHS phase	Children, n	PSUs, n
AGO	2016	AO7	7	2232	330
ALB	2018	AL7	7	146	88
ARM	2016	AM7	7	399	185
AZE	2006	AZ5	5	654	268
BDI	2017	BU7	7	1740	230
BEN	2018	BJ7	7	2131	295
BFA	2021	BF8	8	3573	512
BGD	2022	BD8	8	2701	663
BOL	2008	BO5	5	2540	883
CIV	2021	CI8	8	3163	534
CMR	2019	CM7	7	11	3
COD	2014	CD6	6	605	137
COG	2012	CG6	6	247	61
COM	2012	KM6	6	926	243
DMA	2013	DR6	6	264	106
EGY	2014	EG6	6	5090	1550
ETH	2019	ET7	8	1610	297
GAB	2021	GA7	7	903	184
GHA	2023	GH8	8	322	80
GIN	2018	GN7	7	2157	397
GMB	2020	GM7	7	2151	197
GTM	2015	GU6	6	2461	570
GUY	2009	GY5	5	645	261
HND	2012	HN6	6	1887	578
HTI	2017	HT7	7	1543	355
IDN	2017	ID7	7	5367	1813
IND	2021	IA7	7	16827	6443

ISO3	Survey year	DHS dataset	DHS phase	Children, n	PSUs, n
JOR	2023	JO8	8	2518	841
KEN	2022	KE8	8	5918	1596
KGZ	2012	KY6	6	1398	305
LBR	2020	LB7	7	773	142
LSO	2014	LS6	6	475	268
MDG	2021	MD7	7	3762	644
MDV	2017	MV7	7	702	184
MLI	2018	ML7	7	3010	344
MMR	2016	MM7	7	1120	333
MOZ	2023	MZ8	8	463	96
MRT	2021	MR7	7	377	153
MWI	2016	MW7	7	1578	267
NAM	2013	NM6	6	681	370
NER	2012	NI6	6	1615	457
NGA	2018	NG7	7	9893	1378
NPL	2022	NP8	8	422	139
PAK	2018	PK7	7	2574	402
PER	2012	PE6	6	2781	1151
PHL	2022	PH8	8	2410	1003
PNG	2018	PG7	7	672	181
RWA	2020	RW7	7	1789	359
SEN	2023	SN8	8	3135	398
SLE	2019	SL7	7	3021	570
STP	2009	ST5	5	17	8
SWZ	2007	SZ5	5	158	53
TCD	2015	TD6	6	2062	405
TGO	2014	TG6	6	683	184
TJK	2017	TJ7	7	1929	355

ISO3	Survey year	DHS dataset	DHS phase	Children, n	PSUs, n
TLS	2016	TL7	7	2212	448
TZA	2022	TZ8	8	3345	620
UGA	2016	UG7	7	4707	693
YEM	2013	YE6	6	4602	767
ZAF	2016	ZA7	7	1032	484
ZMB	2019	ZM7	7	73	15
ZWE	2015	ZW7	7	1795	388

DHS - Demographic and Health Surveys; ISO3 - three-letter country code; PSU - primary sampling unit. Table S1 documents the survey inventory and is not a sequential participant-flow table.

Table S2. Country-level missingness of minimum dietary diversity variables.

Country code	Total, n	MDD missing, n	Unweighted missing, %	Weighted missing, %
AL	146	0	0.0	0.0
AM	399	3	0.8	0.6
AO	2232	83	3.7	3.9
AZ	654	9	1.4	2.2
BD	2701	76	2.8	2.8
BF	3573	108	3.0	2.7
BJ	2131	87	4.1	4.2
BO	2540	20	0.8	0.6
BU	1740	65	3.7	3.8
CD	605	9	1.5	1.3
CG	247	1	0.4	0.2
CI	3163	189	6.0	6.2
CM	11	0	0.0	0.0
DR	264	2	0.8	0.9
EG	5090	7	0.1	0.1
ET	1610	92	5.7	4.6
GA	903	58	6.4	5.1
GH	322	12	3.7	3.2
GM	2151	88	4.1	3.7
GN	2157	171	7.9	8.5
GU	2461	6	0.2	0.2
GY	645	29	4.5	5.1
HN	1887	13	0.7	0.6
HT	1543	120	7.8	8.9
IA	16827	537	3.2	3.2
ID	5367	261	4.9	4.2
JO	2518	32	1.3	1.8
KE	5918	2953	49.9	50.8
KM	926	18	1.9	2.1
KY	1398	15	1.1	1.3
LB	773	68	8.8	7.5
LS	475	2	0.4	0.5
MD	3762	151	4.0	4.0
ML	3010	166	5.5	4.5
MM	1120	0	0.0	0.0
MR	377	11	2.9	3.0
MV	702	17	2.4	2.1
MW	1578	64	4.1	4.2
MZ	463	14	3.0	3.2

Country code	Total, n	MDD missing, n	Unweighted missing, %	Weighted missing, %
NG	9893	623	6.3	6.4
NI	1615	9	0.6	0.6
NM	681	10	1.5	1.0
NP	422	5	1.2	1.3
PE	2781	0	0.0	0.0
PG	672	51	7.6	8.1
PH	2410	68	2.8	3.4
PK	1990	101	5.1	6.0
RW	1789	59	3.3	3.3
SL	3021	283	9.4	10.1
SN	3135	117	3.7	3.4
ST	17	0	0.0	0.0
SZ	158	0	0.0	0.0
TD	2062	23	1.1	1.2
TG	683	10	1.5	1.5
TJ	1929	45	2.3	2.3
TL	2212	113	5.1	5.7
TZ	3345	120	3.6	3.2
UG	4707	285	6.1	6.2
YE	4602	81	1.8	1.7
ZA	1032	137	13.3	14.6
ZM	73	3	4.1	4.5
ZW	1795	122	6.8	6.7

MDD - minimum dietary diversity. Table S2 summarises variable availability; its denominator is table-specific and should not be interpreted as the next stage of a sequential participant-flow calculation.

Table S3. Design-adjusted comparison of characteristics between children with missing and non-missing minimum dietary diversity data, by country.

ISO3	Variable	Test	Statistic	df	P
AGO	Sex	Rao-Scott F	0.340	1, 329	0.560
ALB	Sex	NA	NA	NA	NA
ARM	Sex	Rao-Scott F	0.010	1, 184	0.921
AZE	Sex	Rao-Scott F	5.154	1, 267	0.024
BDI	Sex	Rao-Scott F	0.648	1, 229	0.422
BEN	Sex	Rao-Scott F	0.927	1, 294	0.336
BFA	Sex	Rao-Scott F	0.424	1, 511	0.515
BGD	Sex	Rao-Scott F	0.131	1, 662	0.718
BOL	Sex	Rao-Scott F	1.093	1, 882	0.296
CIV	Sex	Rao-Scott F	0.429	1, 533	0.513
CMR	Sex	NA	NA	NA	NA
COD	Sex	Rao-Scott F	0.288	1, 136	0.592
COG	Sex	Rao-Scott F	0.755	1, 60	0.388
COM	Sex	Rao-Scott F	4.537	1, 242	0.034
DMA	Sex	Rao-Scott F	2.361	1, 105	0.127
EGY	Sex	Rao-Scott F	0.373	11, 549	0.541
ETH	Sex	Rao-Scott F	0.936	1, 296	0.334
GAB	Sex	Rao-Scott F	0.039	1, 183	0.844
GHA	Sex	Rao-Scott F	0.411	1, 79	0.523
GIN	Sex	Rao-Scott F	5.928e-05	1, 396	0.994
GMB	Sex	Rao-Scott F	0.734	1, 196	0.393
GTM	Sex	Rao-Scott F	9.259	1, 569	0.002
GUY	Sex	Rao-Scott F	0.349	1, 260	0.555
HND	Sex	Rao-Scott F	0.093	1, 577	0.761
HTI	Sex	Rao-Scott F	1.476	1, 354	0.225
IDN	Sex	Rao-Scott F	0.087	11, 812	0.768
IND	Sex	Rao-Scott F	0.044	16, 442	0.835

ISO3	Variable	Test	Statistic	df	P
JOR	Sex	Rao-Scott F	0.225	1, 840	0.635
KEN	Sex	Rao-Scott F	1.801	11, 595	0.180
KGZ	Sex	Rao-Scott F	0.287	1, 304	0.592
LBR	Sex	Rao-Scott F	0.066	1, 141	0.797
LSO	Sex	Rao-Scott F	0.016	1, 267	0.899
MDG	Sex	Rao-Scott F	0.025	1, 643	0.875
MDV	Sex	Rao-Scott F	0.561	1, 183	0.455
MLI	Sex	Rao-Scott F	0.578	1, 343	0.447
MMR	Sex	NA	NA	NA	NA
MOZ	Sex	Rao-Scott F	1.068	1, 95	0.304
MRT	Sex	Rao-Scott F	0.088	1, 152	0.767
MWI	Sex	Rao-Scott F	0.042	1, 266	0.837
NAM	Sex	Rao-Scott F	0.027	1, 369	0.869
NER	Sex	Rao-Scott F	0.021	1, 456	0.886
NGA	Sex	Rao-Scott F	1.624	11, 377	0.203
NPL	Sex	Rao-Scott F	7.320e-04	1, 138	0.978
PAK	Sex	Rao-Scott F	1.001	1, 312	0.318
PER	Sex	NA	NA	NA	NA
PHL	Sex	Rao-Scott F	0.120	11, 2	0.729
PNG	Sex	Rao-Scott F	4.014	1, 180	0.047
RWA	Sex	Rao-Scott F	1.907	1, 358	0.168
SEN	Sex	Rao-Scott F	2.608	1, 397	0.107
SLE	Sex	Rao-Scott F	1.206	1, 569	0.273
STP	Sex	NA	NA	NA	NA
SWZ	Sex	NA	NA	NA	NA
TCD	Sex	Rao-Scott F	2.532	1, 404	0.112
TGO	Sex	Rao-Scott F	0.788	1, 183	0.376
TJK	Sex	Rao-Scott F	2.792	1, 354	0.096

ISO3	Variable	Test	Statistic	df	P
TLS	Sex	Rao-Scott F	0.648	1, 447	0.421
TZA	Sex	Rao-Scott F	0.074	1, 619	0.785
UGA	Sex	Rao-Scott F	2.129	1, 692	0.145
YEM	Sex	Rao-Scott F	2.153	1, 766	0.143
ZAF	Sex	Rao-Scott F	0.226	1, 483	0.634
ZMB	Sex	Rao-Scott F	0.001	1, 14	0.972
ZWE	Sex	Rao-Scott F	1.612	1, 387	0.205
AGO	Residence	Rao-Scott F	0.370	1, 329	0.543
ALB	Residence	NA	NA	NA	NA
ARM	Residence	Rao-Scott F	4.274	1, 184	0.040
AZE	Residence	Rao-Scott F	14.884	1, 267	<0.001
BDI	Residence	Rao-Scott F	0.277	1, 229	0.599
BEN	Residence	Rao-Scott F	1.182	1, 294	0.278
BFA	Residence	Rao-Scott F	0.126	1, 511	0.723
BGD	Residence	Rao-Scott F	0.562	1, 662	0.454
BOL	Residence	Rao-Scott F	0.071	1, 882	0.790
CIV	Residence	Rao-Scott F	5.432	1, 533	0.020
CMR	Residence	NA	NA	NA	NA
COD	Residence	Rao-Scott F	0.646	1, 136	0.423
COG	Residence	Rao-Scott F	0.827	1, 60	0.367
COM	Residence	Rao-Scott F	17.885	1, 242	<0.001
DMA	Residence	Rao-Scott F	NA	NA	NA
EGY	Residence	Rao-Scott F	0.085	11, 549	0.771
ETH	Residence	Rao-Scott F	1.209	1, 296	0.272
GAB	Residence	Rao-Scott F	5.383	1, 183	0.021
GHA	Residence	Rao-Scott F	2.408	1, 79	0.125
GIN	Residence	Rao-Scott F	0.413	1, 396	0.521
GMB	Residence	Rao-Scott F	0.516	1, 196	0.473

ISO3	Variable	Test	Statistic	df	P
GTM	Residence	Rao-Scott F	3.758	1, 569	0.053
GUY	Residence	Rao-Scott F	0.344	1, 260	0.558
HND	Residence	Rao-Scott F	8.978	1, 577	0.003
HTI	Residence	Rao-Scott F	3.269	1, 354	0.071
IDN	Residence	Rao-Scott F	0.153	11, 812	0.696
IND	Residence	Rao-Scott F	4.202	16, 442	0.040
JOR	Residence	Rao-Scott F	0.146	1, 840	0.702
KEN	Residence	Rao-Scott F	0.590	11, 595	0.443
KGZ	Residence	Rao-Scott F	2.278	1, 304	0.132
LBR	Residence	Rao-Scott F	2.252	1, 141	0.136
LSO	Residence	Rao-Scott F	0.387	1, 267	0.534
MDG	Residence	Rao-Scott F	0.269	1, 643	0.604
MDV	Residence	Rao-Scott F	NA	NA	NA
MLI	Residence	Rao-Scott F	0.523	1, 343	0.470
MMR	Residence	NA	NA	NA	NA
MOZ	Residence	Rao-Scott F	0.465	1, 95	0.497
MRT	Residence	Rao-Scott F	NA	NA	NA
MWI	Residence	Rao-Scott F	0.279	1, 266	0.598
NAM	Residence	Rao-Scott F	0.338	1, 369	0.562
NER	Residence	Rao-Scott F	0.260	1, 456	0.610
NGA	Residence	Rao-Scott F	3.219	11, 377	0.073
NPL	Residence	Rao-Scott F	0.098	1, 138	0.755
PAK	Residence	Rao-Scott F	0.022	1, 312	0.883
PER	Residence	NA	NA	NA	NA
PHL	Residence	Rao-Scott F	3.247	11, 2	0.072
PNG	Residence	Rao-Scott F	10.347	1, 180	0.002
RWA	Residence	Rao-Scott F	1.177e-04	1, 358	0.991
SEN	Residence	Rao-Scott F	0.815	1, 397	0.367

ISO3	Variable	Test	Statistic	df	P
SLE	Residence	Rao-Scott F	4.295	1, 569	0.039
STP	Residence	NA	NA	NA	NA
SWZ	Residence	NA	NA	NA	NA
TCD	Residence	Rao-Scott F	1.238e-04	1, 404	0.991
TGO	Residence	Rao-Scott F	0.034	1, 183	0.854
TJK	Residence	Rao-Scott F	1.523	1, 354	0.218
TLS	Residence	Rao-Scott F	0.910	1, 447	0.341
TZA	Residence	Rao-Scott F	2.286	1, 619	0.131
UGA	Residence	Rao-Scott F	1.509	1, 692	0.220
YEM	Residence	Rao-Scott F	0.458	1, 766	0.499
ZAF	Residence	Rao-Scott F	3.227	1, 483	0.073
ZMB	Residence	Rao-Scott F	0.585	1, 14	0.457
ZWE	Residence	Rao-Scott F	0.137	1, 387	0.711
AGO	Wealth quintile	Rao-Scott F	1.386	3.548, 1167.415	0.241
ALB	Wealth quintile	NA	NA	NA	NA
ARM	Wealth quintile	Rao-Scott F	0.510	2.433, 447.649	0.637
AZE	Wealth quintile	Rao-Scott F	3.122	3.094, 826.148	0.024
BDI	Wealth quintile	Rao-Scott F	3.856	3.825, 875.850	0.005
BEN	Wealth quintile	Rao-Scott F	1.144	3.908, 1149.013	0.334
BFA	Wealth quintile	Rao-Scott F	2.450	3.836, 1960.446	0.047
BGD	Wealth quintile	Rao-Scott F	0.375	3.904, 2584.166	0.822
BOL	Wealth quintile	Rao-Scott F	0.643	3.449, 3042.376	0.609
CIV	Wealth quintile	Rao-Scott F	3.172	3.445, 1835.962	0.018
CMR	Wealth quintile	NA	NA	NA	NA
COD	Wealth quintile	Rao-Scott F	0.882	2.118, 288.053	0.420
COG	Wealth quintile	Rao-Scott F	0.072	3.019, 181.146	0.975
COM	Wealth quintile	Rao-Scott F	1.456	3.718, 899.843	0.217
DMA	Wealth quintile	Rao-Scott F	3.259	2.891, 303.512	0.023

ISO3	Variable	Test	Statistic	df	P
EGY	Wealth quintile	Rao-Scott F	0.871	2.512, 3891.166	0.439
ETH	Wealth quintile	Rao-Scott F	0.407	3.736, 1105.749	0.791
GAB	Wealth quintile	Rao-Scott F	2.654	3.356, 614.089	0.041
GHA	Wealth quintile	Rao-Scott F	1.007	3.600, 284.403	0.399
GIN	Wealth quintile	Rao-Scott F	1.425	3.886, 1538.953	0.224
GMB	Wealth quintile	Rao-Scott F	2.616	3.661, 717.583	0.039
GTM	Wealth quintile	Rao-Scott F	0.562	3.090, 1758.278	0.645
GUY	Wealth quintile	Rao-Scott F	1.189	3.223, 838.047	0.314
HND	Wealth quintile	Rao-Scott F	0.222	3.889, 2243.955	0.922
HTI	Wealth quintile	Rao-Scott F	1.940	3.819, 1352.029	0.105
IDN	Wealth quintile	Rao-Scott F	1.179	3.766, 6823.115	0.318
IND	Wealth quintile	Rao-Scott F	4.589	3.861, 24873.169	0.001
JOR	Wealth quintile	Rao-Scott F	1.052	3.221, 2705.641	0.371
KEN	Wealth quintile	Rao-Scott F	0.593	3.136, 5001.333	0.627
KGZ	Wealth quintile	Rao-Scott F	0.951	2.476, 752.586	0.402
LBR	Wealth quintile	Rao-Scott F	0.753	3.583, 505.230	0.543
LSO	Wealth quintile	Rao-Scott F	0.806	3.748, 1000.726	0.514
MDG	Wealth quintile	Rao-Scott F	0.953	3.968, 2551.279	0.432
MDV	Wealth quintile	Rao-Scott F	0.554	2.938, 537.649	0.642
MLI	Wealth quintile	Rao-Scott F	0.769	3.891, 1334.720	0.542
MMR	Wealth quintile	NA	NA	NA	NA
MOZ	Wealth quintile	Rao-Scott F	1.179	2.734, 259.686	0.317
MRT	Wealth quintile	Rao-Scott F	0.568	2.538, 385.775	0.608
MWI	Wealth quintile	Rao-Scott F	1.557	3.837, 1020.713	0.186
NAM	Wealth quintile	Rao-Scott F	1.135	3.792, 1399.329	0.338
NER	Wealth quintile	Rao-Scott F	0.970	3.333, 1519.761	0.412
NGA	Wealth quintile	Rao-Scott F	3.454	3.959, 5451.838	0.008
NPL	Wealth quintile	Rao-Scott F	0.520	3.889, 536.647	0.716

ISO3	Variable	Test	Statistic	df	P
PAK	Wealth quintile	Rao-Scott F	1.351	3.777, 1178.549	0.251
PER	Wealth quintile	NA	NA	NA	NA
PHL	Wealth quintile	Rao-Scott F	0.752	3.771, 3778.641	0.549
PNG	Wealth quintile	Rao-Scott F	1.497	3.255, 585.903	0.211
RWA	Wealth quintile	Rao-Scott F	0.592	3.928, 1406.188	0.665
SEN	Wealth quintile	Rao-Scott F	1.408	2.980, 1182.898	0.239
SLE	Wealth quintile	Rao-Scott F	1.149	3.922, 2231.497	0.331
STP	Wealth quintile	NA	NA	NA	NA
SWZ	Wealth quintile	NA	NA	NA	NA
TCD	Wealth quintile	Rao-Scott F	1.634	3.802, 1536.163	0.166
TGO	Wealth quintile	Rao-Scott F	2.606	3.649, 667.689	0.040
TJK	Wealth quintile	Rao-Scott F	0.395	3.592, 1271.483	0.792
TLS	Wealth quintile	Rao-Scott F	0.289	3.780, 1689.819	0.876
TZA	Wealth quintile	Rao-Scott F	3.575	3.932, 2434.138	0.007
UGA	Wealth quintile	Rao-Scott F	2.519	3.881, 2685.997	0.041
YEM	Wealth quintile	Rao-Scott F	1.274	3.725, 2853.714	0.279
ZAF	Wealth quintile	Rao-Scott F	0.655	3.888, 1877.688	0.619
ZMB	Wealth quintile	Rao-Scott F	1.300	3.100, 43.404	0.287
ZWE	Wealth quintile	Rao-Scott F	1.077	3.746, 1449.750	0.365

MDD - minimum dietary diversity; NA - not available. P values are from design-adjusted Rao-Scott tests.

Table S4. Country-level survey-weighted prevalence of minimum dietary diversity under the 2021 WHO/UNICEF definition, with 95% confidence intervals.

ISO3	MDD prevalence, %	SE, percentage points	95% CI lower, %	95% CI upper, %
AGO	25.2	2.1	21.1	29.2
ALB	39.7	6.2	27.5	51.9
ARM	37.2	3.2	30.9	43.4
AZE	33.2	2.6	28.2	38.3
BDI	20.0	1.4	17.2	22.7
BEN	26.0	1.7	22.8	29.3
BFA	7.8	0.7	6.5	9.1
BGD	33.8	1.2	31.4	36.2
BOL	63.9	1.3	61.4	66.4
CIV	14.4	1.4	11.7	17.1
CMR	29.7	3.3	23.2	36.2
COD	11.9	2.0	8.1	15.8
COG	9.3	2.4	4.6	14.0
COM	21.4	2.1	17.2	25.6
DMA	37.5	3.2	31.2	43.9
EGY	33.6	0.9	31.9	35.3
ETH	13.2	1.7	9.9	16.5
GAB	14.0	2.1	9.8	18.2
GHA	19.4	3.3	13.0	25.7
GIN	14.2	1.0	12.2	16.2
GMB	21.5	1.5	18.5	24.5
GTM	59.6	1.3	57.0	62.2
GUY	49.5	2.5	44.6	54.3
HND	61.4	1.7	58.0	64.8
HTI	16.8	1.3	14.2	19.5
IDN	55.4	0.9	53.5	57.2
IND	17.7	0.4	16.9	18.6
JOR	35.9	1.6	32.8	39.1
KEN	30.3	1.4	27.7	33.0
KGZ	36.2	2.2	32.0	40.5
LBR	7.3	1.4	4.7	10.0
LSO	14.2	1.7	10.9	17.5
MDG	25.9	1.4	23.3	28.6
MDV	69.2	2.0	65.2	73.1
MLI	21.9	1.4	19.1	24.7
MMR	18.6	1.7	15.4	21.9
MOZ	8.1	1.4	5.3	10.9
MRT	27.5	3.4	20.8	34.1

ISO3	MDD prevalence, %	SE, percentage points	95% CI lower, %	95% CI upper, %
MWI	16.8	1.8	13.3	20.3
NAM	24.4	2.3	19.9	28.9
NER	8.4	0.9	6.7	10.1
NGA	22.5	0.7	21.1	23.9
NPL	36.9	2.9	31.2	42.6
PAK	14.1	1.3	11.6	16.6
PER	68.0	1.3	65.5	70.5
PHL	40.5	1.5	37.5	43.5
PNG	21.8	2.8	16.4	27.3
RWA	37.2	1.5	34.2	40.2
SEN	20.5	1.3	17.9	23.0
SLE	25.2	1.1	23.0	27.4
STP	64.7	11.5	42.2	87.2
SWZ	32.7	3.7	25.5	39.9
TCD	9.0	1.1	6.8	11.2
TGO	16.2	1.7	12.8	19.5
TJK	21.0	1.2	18.7	23.3
TLS	26.4	1.8	22.9	29.9
TZA	12.9	0.9	11.2	14.6
UGA	24.2	1.0	22.4	26.1
YEM	20.6	1.0	18.6	22.5
ZAF	39.8	1.9	36.1	43.5
ZMB	23.3	7.3	9.1	37.6
ZWE	22.8	1.4	20.1	25.6

CI - confidence interval; ISO3 - three-letter country code; MDD - minimum dietary diversity; SE - standard error; UNICEF - United Nations Children's Fund; WHO - World Health Organization.

Table S5. Country-level survey-weighted prevalence of minimum dietary diversity under the 2008 WHO/UNICEF definition, with 95% confidence intervals.

ISO3	MDD prevalence, %	SE, percentage points	95% CI lower, %	95% CI upper, %
AGO	29.0	2.3	24.5	33.5
ALB	49.0	7.1	35.2	62.8
ARM	50.4	3.2	44.1	56.7
AZE	48.0	2.8	42.6	53.4
BDI	22.1	1.5	19.2	25.1
BEN	29.2	1.7	25.9	32.5
BFA	9.6	0.7	8.2	11.0
BGD	35.3	1.2	32.9	37.6
BOL	70.1	1.3	67.6	72.7
CIV	20.0	1.6	16.8	23.1
CMR	29.7	3.3	23.2	36.2
COD	13.6	2.1	9.5	17.7
COG	19.8	3.1	13.7	25.9
COM	24.7	2.3	20.2	29.2
DMA	47.4	3.4	40.7	54.2
EGY	41.9	0.9	40.2	43.6
ETH	14.3	1.8	10.7	17.9
GAB	23.4	2.0	19.5	27.4
GHA	23.8	3.7	16.6	31.0
GIN	16.1	1.1	13.9	18.3
GMB	25.1	1.7	21.9	28.4
GTM	63.5	1.3	60.8	66.1
GUY	58.3	2.8	52.9	63.7
HND	68.8	1.6	65.7	71.8
HTI	22.7	1.6	19.6	25.7
IDN	61.4	0.9	59.6	63.3
IND	20.2	0.5	19.2	21.1
JOR	51.0	1.7	47.7	54.3
KEN	36.2	1.4	33.5	39.0
KGZ	42.8	2.1	38.6	47.0
LBR	7.8	1.4	5.1	10.5
LSO	20.5	2.1	16.3	24.6
MDG	28.3	1.4	25.6	31.0
MDV	72.5	1.8	68.9	76.1
MLI	24.3	1.5	21.3	27.3
MMR	21.7	1.8	18.1	25.2
MOZ	10.8	1.9	7.0	14.5
MRT	35.6	3.4	28.8	42.3

ISO3	MDD prevalence, %	SE, percentage points	95% CI lower, %	95% CI upper, %
MWI	18.9	1.8	15.4	22.5
NAM	31.1	2.5	26.2	36.0
NER	10.0	1.0	8.0	11.9
NGA	27.7	0.8	26.1	29.3
NPL	38.2	3.0	32.4	44.0
PAK	19.6	1.4	16.8	22.5
PER	73.4	1.2	71.1	75.8
PHL	48.3	1.6	45.1	51.5
PNG	24.1	3.1	18.0	30.2
RWA	39.9	1.6	36.8	43.1
SEN	25.5	1.4	22.9	28.2
SLE	29.7	1.2	27.2	32.1
STP	76.0	7.1	62.1	89.8
SWZ	38.3	4.3	29.9	46.7
TCD	10.1	1.2	7.9	12.4
TGO	17.9	1.8	14.5	21.4
TJK	27.2	1.4	24.5	29.9
TLS	31.9	1.8	28.4	35.4
TZA	15.8	1.0	13.9	17.7
UGA	28.6	1.1	26.5	30.6
YEM	25.7	1.1	23.6	27.7
ZAF	49.0	2.1	44.9	53.0
ZMB	26.8	8.2	10.7	42.9
ZWE	29.6	1.6	26.5	32.6

CI - confidence interval; ISO3 - three-letter country code; MDD - minimum dietary diversity; SE - standard error; UNICEF - United Nations Children's Fund; WHO - World Health Organization.

Table S6. Country-level survey-weighted point estimates for four child health outcomes.

ISO3	OWOB, %	Stunting, %	Underweight, %	Anaemia, %
AGO	4.3	38.5	21.3	71.6
ALB	27.0	2.8	0.2	34.2
ARM	16.8	11.2	1.4	21.3
AZE	8.3	15.5	7.4	53.3
BDI	2.0	51.7	28.7	61.6
BEN	2.3	28.6	17.4	79.4
BFA	1.6	22.0	18.9	82.8
BGD	2.4	23.5	18.2	NR
BOL	10.9	23.4	5.0	41.4
CIV	3.5	22.0	16.3	70.9
CMR	NR	NR	NR	66.0
COD	5.4	44.3	23.8	59.1
COG	4.7	27.0	17.9	83.3
COM	12.4	32.6	15.9	NR
DMA	4.6	11.3	6.6	NR
EGY	17.1	21.1	5.5	40.0
ETH	3.5	31.1	19.2	NR
GAB	5.8	22.7	5.6	66.7
GHA	1.7	13.7	10.0	46.6
GIN	5.8	31.1	18.6	79.1
GMB	2.2	22.8	13.7	52.3
GTM	3.6	46.0	13.7	41.6
GUY	8.4	20.3	11.8	58.5
HND	5.8	17.4	5.8	41.7
HTI	2.8	17.5	8.8	79.1
IND	3.9	33.2	26.8	67.9
JOR	11.9	7.0	2.1	38.6

ISO3	OWOB, %	Stunting, %	Underweight, %	Anaemia, %
KEN	4.4	18.8	9.8	NR
KGZ	12.5	15.3	2.2	46.2
LBR	4.6	27.6	10.8	73.0
LSO	9.4	28.6	10.9	45.3
MDG	2.2	39.1	22.8	60.2
MDV	5.7	22.9	11.6	34.8
MLI	1.7	25.4	22.2	86.2
MMR	1.5	21.6	16.5	64.1
MOZ	4.6	31.9	18.4	84.8
MRT	1.5	17.8	10.2	70.1
MWI	4.4	36.6	13.2	76.3
NAM	4.7	16.8	11.7	54.1
NER	2.4	38.6	41.9	79.0
NGA	2.6	33.4	23.2	77.2
PAK	4.7	29.3	16.8	NR
PER	9.8	18.8	4.4	37.8
PNG	10.3	41.5	27.0	NR
RWA	8.5	29.2	7.2	38.3
SEN	1.1	17.7	17.5	NR
SLE	5.6	26.3	16.6	68.3
STP	29.3	9.6	7.5	83.6
SWZ	18.5	26.2	3.7	62.5
TCD	2.2	30.7	27.3	NR
TGO	1.9	22.3	16.6	82.1
TJK	4.4	14.6	9.1	53.1
TLS	8.0	42.9	38.5	45.9
TZA	5.0	30.5	12.7	67.4
UGA	6.6	29.1	12.0	65.7

ISO3	OWOB, %	Stunting, %	Underweight, %	Anaemia, %
YEM	2.7	37.5	35.6	75.2
ZAF	18.8	29.7	5.5	41.3
ZMB	10.3	26.9	7.6	62.5
NPL	NR	26.3	25.2	64.0
ZWE	NR	27.1	11.6	42.7

ISO3 - three-letter country code; NR - not reported; OWOB - overweight/obesity. Confidence intervals were removed because the supplied source contained invalid bounds. For Cameroon, the source output duplicated the OWOB and stunting values and they were not independently verifiable; both values are reported as NR. Cameroon underweight was absent from the source output and is also reported as NR.

Table S7. Associations of binary minimum dietary diversity indicators under the 2021 and 2008 definitions with four child health outcomes.

Framework	Income group	Outcome	Outcome events among MDD achievers, n	aOR	95% CI	P
2021	Upper-middle-income	OWOB	334	1.010	0.818 to 1.248	0.923
2021	Upper-middle-income	Stunting	1257	0.677	0.593 to 0.774	<0.001
2021	Upper-middle-income	Underweight	376	0.695	0.577 to 0.837	<0.001
2021	Upper-middle-income	Anaemia	1966	0.954	0.845 to 1.078	0.452
2021	Lower-middle-income	OWOB	986	1.067	0.954 to 1.194	0.254
2021	Lower-middle-income	Stunting	3472	0.822	0.773 to 0.874	<0.001
2021	Lower-middle-income	Underweight	1977	0.778	0.723 to 0.837	<0.001
2021	Lower-middle-income	Anaemia	5883	0.973	0.910 to 1.041	0.432
2021	Low-income	OWOB	156	1.042	0.828 to 1.310	0.727
2021	Low-income	Stunting	1216	0.731	0.666 to 0.802	<0.001
2021	Low-income	Underweight	811	0.782	0.700 to 0.875	<0.001
2021	Low-income	Anaemia	2088	1.021	0.921 to 1.133	0.692
2008	Upper-middle-income	OWOB	389	1.011	0.812 to 1.260	0.921
2008	Upper-middle-income	Stunting	1404	0.650	0.569 to 0.743	<0.001
2008	Upper-middle-income	Underweight	414	0.656	0.545 to 0.791	<0.001
2008	Upper-middle-income	Anaemia	2215	0.915	0.806 to 1.039	0.169
2008	Lower-middle-income	OWOB	1197	1.118	1.005 to 1.243	0.041
2008	Lower-middle-income	Stunting	4075	0.779	0.734 to 0.827	<0.001
2008	Lower-middle-income	Underweight	2276	0.756	0.705 to 0.811	<0.001
2008	Lower-middle-income	Anaemia	6895	0.969	0.909 to 1.033	0.330
2008	Low-income	OWOB	182	1.075	0.864 to 1.338	0.513
2008	Low-income	Stunting	1434	0.710	0.649 to 0.776	<0.001
2008	Low-income	Underweight	935	0.745	0.671 to 0.828	<0.001
2008	Low-income	Anaemia	2408	1.012	0.917 to 1.117	0.810

aOR - adjusted odds ratio; CI - confidence interval; MDD - minimum dietary diversity; OWOB - overweight/obesity.

Table S8. Associations of continuous minimum dietary diversity scores under the 2021 and 2008 frameworks with four child health outcomes.

Framework	Income group	Outcome	Total, n	Outcome absent, n	Outcome present, n	aOR per 1-point MDD	95% CI	P
2021	Upper-middle-income	OWOB	9240	8544	696	1.022	0.966 to 1.081	0.456
2021	Upper-middle-income	Stunting	9271	6715	2556	0.891	0.859 to 0.924	<0.001
2021	Upper-middle-income	Underweight	9317	8494	823	0.903	0.858 to 0.950	<0.001
2021	Upper-middle-income	Anaemia	9147	5016	4131	1.007	0.974 to 1.041	0.676
2021	Lower-middle-income	OWOB	53423	50341	3082	1.016	0.987 to 1.045	0.279
2021	Lower-middle-income	Stunting	53589	39127	14462	0.942	0.928 to 0.957	<0.001
2021	Lower-middle-income	Underweight	54315	44666	9649	0.932	0.916 to 0.948	<0.001
2021	Lower-middle-income	Anaemia	40547	14593	25954	1.009	0.993 to 1.026	0.271
2021	Low-income	OWOB	22939	22186	753	0.994	0.933 to 1.058	0.849
2021	Low-income	Stunting	22928	15606	7322	0.914	0.892 to 0.937	<0.001
2021	Low-income	Underweight	23053	17701	5352	0.920	0.895 to 0.947	<0.001
2021	Low-income	Anaemia	16159	4796	11363	1.020	0.992 to 1.048	0.157
2008	Upper-middle-income	OWOB	9240	8544	696	1.029	0.969 to 1.093	0.345
2008	Upper-middle-income	Stunting	9271	6715	2556	0.873	0.842 to 0.905	<0.001
2008	Upper-middle-income	Underweight	9317	8494	823	0.890	0.848 to 0.935	<0.001
2008	Upper-middle-income	Anaemia	9147	5016	4131	0.987	0.955 to 1.021	0.454
2008	Lower-middle-income	OWOB	53423	50341	3082	1.016	0.987 to 1.045	0.280
2008	Lower-middle-income	Stunting	53589	39127	14462	0.928	0.914 to 0.942	<0.001
2008	Lower-middle-income	Underweight	54315	44666	9649	0.919	0.903 to 0.935	<0.001
2008	Lower-middle-income	Anaemia	40547	14593	25954	0.999	0.982 to 1.016	0.917
2008	Low-income	OWOB	22939	22186	753	0.996	0.933 to 1.064	0.916
2008	Low-income	Stunting	22928	15606	7322	0.899	0.877 to 0.921	<0.001
2008	Low-income	Underweight	23053	17701	5352	0.901	0.876 to 0.927	<0.001
2008	Low-income	Anaemia	16159	4796	11363	1.016	0.989 to 1.045	0.248

aOR - adjusted odds ratio per 1-point increase; CI - confidence interval; MDD - minimum dietary diversity; OWOB - overweight/obesity.

Table S9. Associations of fixed three-category minimum dietary diversity under the 2021 framework with four child health outcomes.

Framework	Income group	Outcome	Category	MDD score range	aOR	95% CI	P
2021	Upper-middle-income	OWOB	Low (reference)	0–2	1.000 (reference)	-	-
2021	Upper-middle-income	Stunting	Low (reference)	0–2	1.000 (reference)	-	-
2021	Upper-middle-income	Underweight	Low (reference)	0–2	1.000 (reference)	-	-
2021	Upper-middle-income	Anaemia	Low (reference)	0–2	1.000 (reference)	-	-
2021	Lower-middle-income	OWOB	Low (reference)	0–2	1.000 (reference)	-	-
2021	Lower-middle-income	Stunting	Low (reference)	0–2	1.000 (reference)	-	-
2021	Lower-middle-income	Underweight	Low (reference)	0–2	1.000 (reference)	-	-
2021	Lower-middle-income	Anaemia	Low (reference)	0–2	1.000 (reference)	-	-
2021	Low-income	OWOB	Low (reference)	0–2	1.000 (reference)	-	-
2021	Low-income	Stunting	Low (reference)	0–2	1.000 (reference)	-	-
2021	Low-income	Underweight	Low (reference)	0–2	1.000 (reference)	-	-
2021	Low-income	Anaemia	Low (reference)	0–2	1.000 (reference)	-	-
2021	Upper-middle-income	OWOB	Medium	3–5	0.990	0.771 to 1.271	0.935
2021	Upper-middle-income	Stunting	Medium	3–5	0.699	0.601 to 0.812	<0.001
2021	Upper-middle-income	Underweight	Medium	3–5	0.691	0.556 to 0.859	<0.001
2021	Upper-middle-income	Anaemia	Medium	3–5	1.073	0.932 to 1.236	0.326
2021	Lower-middle-income	OWOB	Medium	3–5	0.995	0.897 to 1.104	0.922
2021	Lower-middle-income	Stunting	Medium	3–5	0.805	0.762 to 0.850	<0.001
2021	Lower-middle-income	Underweight	Medium	3–5	0.792	0.742 to 0.846	<0.001
2021	Lower-middle-income	Anaemia	Medium	3–5	1.016	0.957 to 1.079	0.607
2021	Low-income	OWOB	Medium	3–5	1.141	0.932 to 1.396	0.202
2021	Low-income	Stunting	Medium	3–5	0.735	0.683 to 0.792	<0.001
2021	Low-income	Underweight	Medium	3–5	0.751	0.689 to 0.818	<0.001
2021	Low-income	Anaemia	Medium	3–5	1.044	0.960 to 1.136	0.315
2021	Upper-middle-income	OWOB	High	6–8	1.051	0.766 to 1.442	0.757
2021	Upper-middle-income	Stunting	High	6–8	0.495	0.402 to 0.610	<0.001
2021	Upper-middle-income	Underweight	High	6–8	0.549	0.413 to 0.730	<0.001

Framework	Income group	Outcome	Category	MDD score range	aOR	95% CI	P
2021	Upper-middle-income	Anaemia	High	6–8	0.939	0.780 to 1.130	0.504
2021	Lower-middle-income	OWOB	High	6–8	1.124	0.936 to 1.350	0.212
2021	Lower-middle-income	Stunting	High	6–8	0.692	0.623 to 0.770	<0.001
2021	Lower-middle-income	Underweight	High	6–8	0.672	0.593 to 0.762	<0.001
2021	Lower-middle-income	Anaemia	High	6–8	0.941	0.836 to 1.059	0.310
2021	Low-income	OWOB	High	6–8	0.805	0.469 to 1.379	0.429
2021	Low-income	Stunting	High	6–8	0.526	0.428 to 0.648	<0.001
2021	Low-income	Underweight	High	6–8	0.518	0.404 to 0.664	<0.001
2021	Low-income	Anaemia	High	6–8	1.083	0.869 to 1.350	0.477

aOR - adjusted odds ratio; CI - confidence interval; MDD - minimum dietary diversity; OWOB - overweight/obesity. The table is restricted to the prespecified 2021 fixed-category analysis.

Table S10. Associations of survey-weighted tertiles of minimum dietary diversity under the 2021 and 2008 frameworks with four child health outcomes.

Framework	Income group	Outcome	Category	MDD score range	aOR	95% CI	P
2021	Upper-middle-income	OWOB	T1 (reference)	0–4	1.000 (reference)	-	-
2021	Upper-middle-income	Stunting	T1 (reference)	0–4	1.000 (reference)	-	-
2021	Upper-middle-income	Underweight	T1 (reference)	0–4	1.000 (reference)	-	-
2021	Upper-middle-income	Anaemia	T1 (reference)	0–4	1.000 (reference)	-	-
2021	Lower-middle-income	OWOB	T1 (reference)	0–2	1.000 (reference)	-	-
2021	Lower-middle-income	Stunting	T1 (reference)	0–2	1.000 (reference)	-	-
2021	Lower-middle-income	Underweight	T1 (reference)	0–2	1.000 (reference)	-	-
2021	Lower-middle-income	Anaemia	T1 (reference)	0–2	1.000 (reference)	-	-
2021	Low-income	OWOB	T1 (reference)	0–2	1.000 (reference)	-	-
2021	Low-income	Stunting	T1 (reference)	0–2	1.000 (reference)	-	-
2021	Low-income	Underweight	T1 (reference)	0–2	1.000 (reference)	-	-
2021	Low-income	Anaemia	T1 (reference)	0–2	1.000 (reference)	-	-
2021	Upper-middle-income	OWOB	T2	5–5	0.884	0.666 to 1.175	0.397
2021	Upper-middle-income	Stunting	T2	5–5	0.716	0.612 to 0.837	<0.001
2021	Upper-middle-income	Underweight	T2	5–5	0.685	0.542 to 0.866	0.002
2021	Upper-middle-income	Anaemia	T2	5–6	0.952	0.837 to 1.083	0.458
2021	Lower-middle-income	OWOB	T2	3–4	0.999	0.893 to 1.117	0.981
2021	Lower-middle-income	Stunting	T2	3–4	0.888	0.838 to 0.942	<0.001
2021	Lower-middle-income	Underweight	T2	3–4	0.900	0.842 to 0.962	0.002
2021	Lower-middle-income	Anaemia	T2	3–4	1.084	1.017 to 1.156	0.013
2021	Low-income	OWOB	T2	3–4	1.007	0.814 to 1.246	0.948
2021	Low-income	Stunting	T2	3–4	0.879	0.810 to 0.954	0.002
2021	Low-income	Underweight	T2	3–4	0.852	0.776 to 0.937	<0.001
2021	Low-income	Anaemia	T2	3–4	1.032	0.935 to 1.138	0.532
2021	Upper-middle-income	OWOB	T3	6–8	1.099	0.864 to 1.398	0.443
2021	Upper-middle-income	Stunting	T3	6–8	0.655	0.559 to 0.767	<0.001
2021	Upper-middle-income	Underweight	T3	6–8	0.701	0.564 to 0.870	0.001

Framework	Income group	Outcome	Category	MDD score range	aOR	95% CI	P
2021	Upper-middle-income	Anaemia	T3	7–8	0.960	0.807 to 1.141	0.641
2021	Lower-middle-income	OWOB	T3	5–8	1.067	0.939 to 1.212	0.323
2021	Lower-middle-income	Stunting	T3	5–8	0.768	0.715 to 0.824	<0.001
2021	Lower-middle-income	Underweight	T3	5–8	0.734	0.677 to 0.797	<0.001
2021	Lower-middle-income	Anaemia	T3	5–8	1.019	0.943 to 1.101	0.629
2021	Low-income	OWOB	T3	5–8	1.046	0.801 to 1.367	0.740
2021	Low-income	Stunting	T3	5–8	0.676	0.608 to 0.752	<0.001
2021	Low-income	Underweight	T3	5–8	0.714	0.630 to 0.809	<0.001
2021	Low-income	Anaemia	T3	5–8	1.041	0.928 to 1.169	0.491
2008	Upper-middle-income	OWOB	T1 (reference)	0–3	1.000 (reference)	-	-
2008	Upper-middle-income	Stunting	T1 (reference)	0–3	1.000 (reference)	-	-
2008	Upper-middle-income	Underweight	T1 (reference)	0–3	1.000 (reference)	-	-
2008	Upper-middle-income	Anaemia	T1 (reference)	0–3	1.000 (reference)	-	-
2008	Lower-middle-income	OWOB	T1 (reference)	0–2	1.000 (reference)	-	-
2008	Lower-middle-income	Stunting	T1 (reference)	0–2	1.000 (reference)	-	-
2008	Lower-middle-income	Underweight	T1 (reference)	0–2	1.000 (reference)	-	-
2008	Lower-middle-income	Anaemia	T1 (reference)	0–2	1.000 (reference)	-	-
2008	Low-income	OWOB	T1 (reference)	0–2	1.000 (reference)	-	-
2008	Low-income	Stunting	T1 (reference)	0–2	1.000 (reference)	-	-
2008	Low-income	Underweight	T1 (reference)	0–2	1.000 (reference)	-	-
2008	Low-income	Anaemia	T1 (reference)	0–2	1.000 (reference)	-	-
2008	Upper-middle-income	OWOB	T2	4–5	0.992	0.782 to 1.259	0.948
2008	Upper-middle-income	Stunting	T2	4–5	0.709	0.616 to 0.815	<0.001
2008	Upper-middle-income	Underweight	T2	4–5	0.688	0.561 to 0.842	<0.001
2008	Upper-middle-income	Anaemia	T2	4–5	0.941	0.825 to 1.075	0.370
2008	Lower-middle-income	OWOB	T2	3–3	0.906	0.788 to 1.041	0.163
2008	Lower-middle-income	Stunting	T2	3–3	0.858	0.804 to 0.917	<0.001
2008	Lower-middle-income	Underweight	T2	3–3	0.850	0.784 to 0.922	<0.001

Framework	Income group	Outcome	Category	MDD score range	aOR	95% CI	P
2008	Lower-middle-income	Anaemia	T2	3–3	1.040	0.967 to 1.119	0.288
2008	Low-income	OWOB	T2	3–3	1.114	0.873 to 1.421	0.386
2008	Low-income	Stunting	T2	3–3	0.783	0.716 to 0.856	<0.001
2008	Low-income	Underweight	T2	3–3	0.773	0.698 to 0.855	<0.001
2008	Low-income	Anaemia	T2	3–3	1.062	0.958 to 1.176	0.252
2008	Upper-middle-income	OWOB	T3	6–7	1.055	0.797 to 1.397	0.709
2008	Upper-middle-income	Stunting	T3	6–7	0.538	0.445 to 0.651	<0.001
2008	Upper-middle-income	Underweight	T3	6–7	0.592	0.458 to 0.765	<0.001
2008	Upper-middle-income	Anaemia	T3	6–7	0.858	0.726 to 1.014	0.072
2008	Lower-middle-income	OWOB	T3	4–7	1.081	0.966 to 1.211	0.176
2008	Lower-middle-income	Stunting	T3	4–7	0.741	0.695 to 0.789	<0.001
2008	Lower-middle-income	Underweight	T3	4–7	0.720	0.669 to 0.776	<0.001
2008	Lower-middle-income	Anaemia	T3	4–7	0.981	0.916 to 1.051	0.588
2008	Low-income	OWOB	T3	4–7	1.115	0.880 to 1.412	0.368
2008	Low-income	Stunting	T3	4–7	0.658	0.599 to 0.721	<0.001
2008	Low-income	Underweight	T3	4–7	0.692	0.620 to 0.772	<0.001
2008	Low-income	Anaemia	T3	4–7	1.032	0.930 to 1.146	0.547

aOR - adjusted odds ratio; CI - confidence interval; MDD - minimum dietary diversity; OWOB - overweight/obesity; T1-T3 - survey-weighted tertiles.

Table S11. Age-stratified associations between continuous minimum dietary diversity score and four child health outcomes.

Income group	Age, months	Outcome	Exposure	aOR	95% CI	P
Upper-middle-income	6–11	OWOB	Continuous score	1.054	0.959 to 1.158	0.278
Upper-middle-income	6–11	Stunting	Continuous score	0.954	0.895 to 1.017	0.152
Upper-middle-income	6–11	Underweight	Continuous score	0.911	0.831 to 1.000	0.049
Upper-middle-income	6–11	Anaemia	Continuous score	1.044	0.989 to 1.102	0.117
Upper-middle-income	12–17	OWOB	Continuous score	0.979	0.880 to 1.088	0.692
Upper-middle-income	12–17	Stunting	Continuous score	0.856	0.803 to 0.912	<0.001
Upper-middle-income	12–17	Underweight	Continuous score	0.899	0.824 to 0.981	0.017
Upper-middle-income	12–17	Anaemia	Continuous score	0.971	0.916 to 1.028	0.312
Upper-middle-income	18–23	OWOB	Continuous score	1.016	0.923 to 1.117	0.748
Upper-middle-income	18–23	Stunting	Continuous score	0.866	0.817 to 0.917	<0.001
Upper-middle-income	18–23	Underweight	Continuous score	0.887	0.817 to 0.962	0.004
Upper-middle-income	18–23	Anaemia	Continuous score	0.977	0.924 to 1.032	0.401
Lower-middle-income	6–11	OWOB	Continuous score	1.035	0.985 to 1.088	0.175
Lower-middle-income	6–11	Stunting	Continuous score	0.943	0.914 to 0.972	<0.001
Lower-middle-income	6–11	Underweight	Continuous score	0.924	0.892 to 0.957	<0.001
Lower-middle-income	6–11	Anaemia	Continuous score	1.006	0.976 to 1.038	0.693
Lower-middle-income	12–17	OWOB	Continuous score	0.975	0.927 to 1.025	0.324
Lower-middle-income	12–17	Stunting	Continuous score	0.948	0.923 to 0.973	<0.001
Lower-middle-income	12–17	Underweight	Continuous score	0.926	0.900 to 0.953	<0.001
Lower-middle-income	12–17	Anaemia	Continuous score	0.999	0.970 to 1.028	0.919
Lower-middle-income	18–23	OWOB	Continuous score	1.049	0.999 to 1.100	0.053
Lower-middle-income	18–23	Stunting	Continuous score	0.944	0.922 to 0.968	<0.001
Lower-middle-income	18–23	Underweight	Continuous score	0.944	0.918 to 0.971	<0.001
Lower-middle-income	18–23	Anaemia	Continuous score	0.988	0.960 to 1.016	0.402
Low-income	6–11	OWOB	Continuous score	1.000	0.886 to 1.129	0.996
Low-income	6–11	Stunting	Continuous score	0.924	0.878 to 0.972	0.002
Low-income	6–11	Underweight	Continuous score	0.949	0.902 to 0.999	0.047

**Income group**	**Age, months**	**Outcome**	**Exposure**	**aOR**	**95% CI**	**P**
Low-income	6–11	Anaemia	Continuous score	1.055	1.004 to 1.108	0.035
Low-income	12–17	OWOB	Continuous score	1.046	0.959 to 1.141	0.311
Low-income	12–17	Stunting	Continuous score	0.908	0.873 to 0.945	<0.001
Low-income	12–17	Underweight	Continuous score	0.895	0.854 to 0.938	<0.001
Low-income	12–17	Anaemia	Continuous score	0.992	0.946 to 1.040	0.743
Low-income	18–23	OWOB	Continuous score	0.912	0.815 to 1.020	0.107
Low-income	18–23	Stunting	Continuous score	0.918	0.885 to 0.952	<0.001
Low-income	18–23	Underweight	Continuous score	0.925	0.883 to 0.970	0.001
Low-income	18–23	Anaemia	Continuous score	0.959	0.909 to 1.011	0.123

aOR - adjusted odds ratio; CI - confidence interval; MDD - minimum dietary diversity; OWOB - overweight/obesity.

Table S12. Age-stratified associations between minimum dietary diversity achievement and four child health outcomes.

Income group	Age, months	Outcome	Exposure	aOR	95% CI	P
Upper-middle-income	6–11	OWOB	Binary achievement	1.127	0.804 to 1.578	0.487
Upper-middle-income	6–11	Stunting	Binary achievement	0.863	0.683 to 1.090	0.215
Upper-middle-income	6–11	Underweight	Binary achievement	0.714	0.499 to 1.021	0.065
Upper-middle-income	6–11	Anaemia	Binary achievement	1.160	0.943 to 1.426	0.161
Upper-middle-income	12–17	OWOB	Binary achievement	0.885	0.596 to 1.315	0.546
Upper-middle-income	12–17	Stunting	Binary achievement	0.586	0.466 to 0.737	<0.001
Upper-middle-income	12–17	Underweight	Binary achievement	0.654	0.477 to 0.897	0.009
Upper-middle-income	12–17	Anaemia	Binary achievement	0.822	0.669 to 1.010	0.062
Upper-middle-income	18–23	OWOB	Binary achievement	0.992	0.692 to 1.423	0.966
Upper-middle-income	18–23	Stunting	Binary achievement	0.642	0.518 to 0.794	<0.001
Upper-middle-income	18–23	Underweight	Binary achievement	0.697	0.522 to 0.932	0.015
Upper-middle-income	18–23	Anaemia	Binary achievement	0.833	0.676 to 1.027	0.087
Lower-middle-income	6–11	OWOB	Binary achievement	1.064	0.857 to 1.322	0.573
Lower-middle-income	6–11	Stunting	Binary achievement	0.833	0.730 to 0.950	0.006
Lower-middle-income	6–11	Underweight	Binary achievement	0.852	0.726 to 0.999	0.049
Lower-middle-income	6–11	Anaemia	Binary achievement	0.961	0.841 to 1.098	0.556
Lower-middle-income	12–17	OWOB	Binary achievement	1.017	0.853 to 1.212	0.855
Lower-middle-income	12–17	Stunting	Binary achievement	0.821	0.744 to 0.906	<0.001
Lower-middle-income	12–17	Underweight	Binary achievement	0.728	0.648 to 0.817	<0.001
Lower-middle-income	12–17	Anaemia	Binary achievement	0.937	0.838 to 1.048	0.252
Lower-middle-income	18–23	OWOB	Binary achievement	1.142	0.947 to 1.377	0.164
Lower-middle-income	18–23	Stunting	Binary achievement	0.845	0.767 to 0.930	<0.001
Lower-middle-income	18–23	Underweight	Binary achievement	0.795	0.709 to 0.892	<0.001
Lower-middle-income	18–23	Anaemia	Binary achievement	0.946	0.848 to 1.055	0.320
Low-income	6–11	OWOB	Binary achievement	1.381	0.907 to 2.104	0.132
Low-income	6–11	Stunting	Binary achievement	0.793	0.645 to 0.974	0.027
Low-income	6–11	Underweight	Binary achievement	0.796	0.640 to 0.989	0.040

Income group	Age, months	Outcome	Exposure	aOR	95% CI	P
Low-income	6–11	Anaemia	Binary achievement	1.107	0.913 to 1.342	0.300
Low-income	12–17	OWOB	Binary achievement	1.089	0.773 to 1.536	0.625
Low-income	12–17	Stunting	Binary achievement	0.707	0.612 to 0.817	<0.001
Low-income	12–17	Underweight	Binary achievement	0.746	0.630 to 0.884	<0.001
Low-income	12–17	Anaemia	Binary achievement	0.960	0.803 to 1.148	0.656
Low-income	18–23	OWOB	Binary achievement	0.752	0.478 to 1.185	0.219
Low-income	18–23	Stunting	Binary achievement	0.736	0.637 to 0.850	<0.001
Low-income	18–23	Underweight	Binary achievement	0.812	0.675 to 0.977	0.027
Low-income	18–23	Anaemia	Binary achievement	0.906	0.749 to 1.096	0.311

aOR - adjusted odds ratio; CI - confidence interval; MDD - minimum dietary diversity; OWOB - overweight/obesity.

Table S13. Age-stratified associations between fixed minimum dietary diversity categories and four child health outcomes.

Income group	Age, months	Outcome	Contrast	aOR	95% CI	P
Upper-middle-income	6–11	OWOB	Medium (3–5) vs Low (0–2)	1.077	0.709 to 1.635	0.729
Upper-middle-income	6–11	OWOB	High (6–8) vs Low (0–2)	1.447	0.889 to 2.356	0.137
Upper-middle-income	6–11	Stunting	Medium (3–5) vs Low (0–2)	0.901	0.689 to 1.177	0.443
Upper-middle-income	6–11	Stunting	High (6–8) vs Low (0–2)	0.823	0.584 to 1.160	0.266
Upper-middle-income	6–11	Underweight	Medium (3–5) vs Low (0–2)	0.793	0.533 to 1.181	0.253
Upper-middle-income	6–11	Underweight	High (6–8) vs Low (0–2)	0.616	0.366 to 1.039	0.069
Upper-middle-income	6–11	Anaemia	Medium (3–5) vs Low (0–2)	1.157	0.909 to 1.472	0.237
Upper-middle-income	6–11	Anaemia	High (6–8) vs Low (0–2)	1.297	0.976 to 1.723	0.073
Upper-middle-income	12–17	OWOB	Medium (3–5) vs Low (0–2)	0.851	0.508 to 1.428	0.542
Upper-middle-income	12–17	OWOB	High (6–8) vs Low (0–2)	0.848	0.461 to 1.557	0.594
Upper-middle-income	12–17	Stunting	Medium (3–5) vs Low (0–2)	0.741	0.536 to 1.025	0.070
Upper-middle-income	12–17	Stunting	High (6–8) vs Low (0–2)	0.523	0.367 to 0.745	<0.001
Upper-middle-income	12–17	Underweight	Medium (3–5) vs Low (0–2)	0.890	0.538 to 1.472	0.649
Upper-middle-income	12–17	Underweight	High (6–8) vs Low (0–2)	0.809	0.475 to 1.379	0.436
Upper-middle-income	12–17	Anaemia	Medium (3–5) vs Low (0–2)	1.214	0.906 to 1.628	0.194
Upper-middle-income	12–17	Anaemia	High (6–8) vs Low (0–2)	1.059	0.762 to 1.473	0.731
Upper-middle-income	18–23	OWOB	Medium (3–5) vs Low (0–2)	1.290	0.759 to 2.192	0.346
Upper-middle-income	18–23	OWOB	High (6–8) vs Low (0–2)	1.362	0.776 to 2.393	0.282
Upper-middle-income	18–23	Stunting	Medium (3–5) vs Low (0–2)	0.580	0.434 to 0.775	<0.001
Upper-middle-income	18–23	Stunting	High (6–8) vs Low (0–2)	0.450	0.326 to 0.621	<0.001
Upper-middle-income	18–23	Underweight	Medium (3–5) vs Low (0–2)	0.572	0.373 to 0.875	0.010
Upper-middle-income	18–23	Underweight	High (6–8) vs Low (0–2)	0.461	0.298 to 0.715	<0.001
Upper-middle-income	18–23	Anaemia	Medium (3–5) vs Low (0–2)	0.962	0.720 to 1.286	0.795
Upper-middle-income	18–23	Anaemia	High (6–8) vs Low (0–2)	0.940	0.687 to 1.287	0.701
Lower-middle-income	6–11	OWOB	Medium (3–5) vs Low (0–2)	1.144	0.952 to 1.374	0.152
Lower-middle-income	6–11	OWOB	High (6–8) vs Low (0–2)	1.141	0.842 to 1.547	0.394
Lower-middle-income	6–11	Stunting	Medium (3–5) vs Low (0–2)	0.812	0.733 to 0.900	<0.001

Income group	Age, months	Outcome	Contrast	aOR	95% CI	P
Lower-middle-income	6–11	Stunting	High (6–8) vs Low (0–2)	0.836	0.695 to 1.005	0.056
Lower-middle-income	6–11	Underweight	Medium (3–5) vs Low (0–2)	0.788	0.708 to 0.878	<0.001
Lower-middle-income	6–11	Underweight	High (6–8) vs Low (0–2)	0.735	0.580 to 0.932	0.011
Lower-middle-income	6–11	Anaemia	Medium (3–5) vs Low (0–2)	1.076	0.974 to 1.188	0.149
Lower-middle-income	6–11	Anaemia	High (6–8) vs Low (0–2)	0.929	0.766 to 1.127	0.457
Lower-middle-income	12–17	OWOB	Medium (3–5) vs Low (0–2)	0.867	0.716 to 1.049	0.143
Lower-middle-income	12–17	OWOB	High (6–8) vs Low (0–2)	0.835	0.642 to 1.084	0.175
Lower-middle-income	12–17	Stunting	Medium (3–5) vs Low (0–2)	0.882	0.798 to 0.974	0.013
Lower-middle-income	12–17	Stunting	High (6–8) vs Low (0–2)	0.744	0.645 to 0.858	<0.001
Lower-middle-income	12–17	Underweight	Medium (3–5) vs Low (0–2)	0.831	0.745 to 0.926	<0.001
Lower-middle-income	12–17	Underweight	High (6–8) vs Low (0–2)	0.695	0.592 to 0.816	<0.001
Lower-middle-income	12–17	Anaemia	Medium (3–5) vs Low (0–2)	1.005	0.900 to 1.122	0.931
Lower-middle-income	12–17	Anaemia	High (6–8) vs Low (0–2)	0.967	0.827 to 1.130	0.673
Lower-middle-income	18–23	OWOB	Medium (3–5) vs Low (0–2)	1.086	0.903 to 1.305	0.381
Lower-middle-income	18–23	OWOB	High (6–8) vs Low (0–2)	1.219	0.956 to 1.555	0.110
Lower-middle-income	18–23	Stunting	Medium (3–5) vs Low (0–2)	0.910	0.828 to 1.000	0.050
Lower-middle-income	18–23	Stunting	High (6–8) vs Low (0–2)	0.756	0.659 to 0.867	<0.001
Lower-middle-income	18–23	Underweight	Medium (3–5) vs Low (0–2)	1.004	0.897 to 1.125	0.942
Lower-middle-income	18–23	Underweight	High (6–8) vs Low (0–2)	0.747	0.632 to 0.882	<0.001
Lower-middle-income	18–23	Anaemia	Medium (3–5) vs Low (0–2)	1.005	0.901 to 1.121	0.928
Lower-middle-income	18–23	Anaemia	High (6–8) vs Low (0–2)	0.906	0.773 to 1.063	0.227
Low-income	6–11	OWOB	Medium (3–5) vs Low (0–2)	0.865	0.605 to 1.236	0.425
Low-income	6–11	OWOB	High (6–8) vs Low (0–2)	1.389	0.747 to 2.584	0.299
Low-income	6–11	Stunting	Medium (3–5) vs Low (0–2)	0.802	0.695 to 0.926	0.003
Low-income	6–11	Stunting	High (6–8) vs Low (0–2)	0.905	0.646 to 1.268	0.561
Low-income	6–11	Underweight	Medium (3–5) vs Low (0–2)	0.853	0.729 to 1.000	0.049
Low-income	6–11	Underweight	High (6–8) vs Low (0–2)	0.868	0.613 to 1.230	0.427
Low-income	6–11	Anaemia	Medium (3–5) vs Low (0–2)	1.072	0.915 to 1.255	0.390

Income group	Age, months	Outcome	Contrast	aOR	95% CI	P
Low-income	6–11	Anaemia	High (6–8) vs Low (0–2)	1.180	0.869 to 1.602	0.289
Low-income	12–17	OWOB	Medium (3–5) vs Low (0–2)	1.265	0.884 to 1.810	0.198
Low-income	12–17	OWOB	High (6–8) vs Low (0–2)	1.107	0.626 to 1.959	0.726
Low-income	12–17	Stunting	Medium (3–5) vs Low (0–2)	0.865	0.761 to 0.984	0.028
Low-income	12–17	Stunting	High (6–8) vs Low (0–2)	0.601	0.469 to 0.770	<0.001
Low-income	12–17	Underweight	Medium (3–5) vs Low (0–2)	0.790	0.685 to 0.912	0.001
Low-income	12–17	Underweight	High (6–8) vs Low (0–2)	0.523	0.388 to 0.706	<0.001
Low-income	12–17	Anaemia	Medium (3–5) vs Low (0–2)	0.884	0.749 to 1.042	0.141
Low-income	12–17	Anaemia	High (6–8) vs Low (0–2)	1.170	0.882 to 1.551	0.276
Low-income	18–23	OWOB	Medium (3–5) vs Low (0–2)	0.957	0.662 to 1.383	0.814
Low-income	18–23	OWOB	High (6–8) vs Low (0–2)	0.543	0.265 to 1.113	0.095
Low-income	18–23	Stunting	Medium (3–5) vs Low (0–2)	0.871	0.763 to 0.994	0.040
Low-income	18–23	Stunting	High (6–8) vs Low (0–2)	0.620	0.492 to 0.781	<0.001
Low-income	18–23	Underweight	Medium (3–5) vs Low (0–2)	0.885	0.760 to 1.031	0.116
Low-income	18–23	Underweight	High (6–8) vs Low (0–2)	0.636	0.463 to 0.872	0.005
Low-income	18–23	Anaemia	Medium (3–5) vs Low (0–2)	0.936	0.780 to 1.123	0.475
Low-income	18–23	Anaemia	High (6–8) vs Low (0–2)	0.700	0.518 to 0.945	0.020

aOR - adjusted odds ratio; CI - confidence interval; MDD - minimum dietary diversity; OWOB - overweight/obesity.

Table S14. Country-specific design-adjusted odds ratios used in the first-stage individual participant data meta-analysis.

Outcome	Country	Design-adjusted OR	95% CI
Anaemia	Albania	0.542	0.260 to 1.127
Anaemia	Armenia	0.917	0.488 to 1.724
Anaemia	Angola	0.756	0.528 to 1.082
Anaemia	Azerbaijan	0.844	0.536 to 1.328
Anaemia	Burkina Faso	0.738	0.478 to 1.140
Anaemia	Benin	0.900	0.612 to 1.322
Anaemia	Bolivia	1.006	0.697 to 1.451
Anaemia	Burundi	0.717	0.464 to 1.106
Anaemia	Congo - Kinshasa	1.180	0.566 to 2.459
Anaemia	Congo - Brazzaville	0.882	0.198 to 3.933
Anaemia	Côte d’Ivoire	0.436	0.268 to 0.710
Anaemia	Egypt	1.139	0.884 to 1.468
Anaemia	Gabon	0.812	0.408 to 1.615
Anaemia	Ghana	0.574	0.153 to 2.156
Anaemia	Gambia	0.951	0.657 to 1.376
Anaemia	Guinea	0.811	0.527 to 1.248
Anaemia	Guatemala	0.894	0.720 to 1.111
Anaemia	Guyana	0.857	0.540 to 1.359
Anaemia	Honduras	0.905	0.683 to 1.199
Anaemia	Haiti	1.293	0.805 to 2.077
Anaemia	India	0.998	0.886 to 1.125
Anaemia	Jordan	1.050	0.739 to 1.492
Anaemia	Kyrgyzstan	0.989	0.708 to 1.382
Anaemia	Liberia	1.058	0.368 to 3.046
Anaemia	Lesotho	0.864	0.463 to 1.612
Anaemia	Madagascar	0.939	0.722 to 1.220
Anaemia	Mali	1.108	0.710 to 1.730
Anaemia	Myanmar (Burma)	0.916	0.584 to 1.436
Anaemia	Mauritania	2.119	0.848 to 5.298
Anaemia	Maldives	0.689	0.463 to 1.026
Anaemia	Malawi	1.177	0.651 to 2.129
Anaemia	Mozambique	0.516	0.108 to 2.458
Anaemia	Nigeria	0.881	0.715 to 1.086
Anaemia	Niger	1.185	0.732 to 1.917
Anaemia	Namibia	1.067	0.692 to 1.645
Anaemia	Nepal	0.491	0.266 to 0.907
Anaemia	Peru	1.266	0.997 to 1.609
Anaemia	Rwanda	0.855	0.626 to 1.168

Outcome	Country	Design-adjusted OR	95% CI
Anaemia	Sierra Leone	1.134	0.840 to 1.530
Anaemia	Eswatini	1.671	0.746 to 3.741
Anaemia	Togo	1.182	0.607 to 2.302
Anaemia	Tajikistan	1.072	0.829 to 1.385
Anaemia	Timor-Leste	1.098	0.766 to 1.574
Anaemia	Tanzania	0.917	0.587 to 1.431
Anaemia	Uganda	1.241	0.915 to 1.683
Anaemia	Yemen	1.006	0.682 to 1.485
Anaemia	South Africa	0.798	0.466 to 1.368
Anaemia	Zambia	0.849	0.244 to 2.952
Anaemia	Zimbabwe	1.062	0.817 to 1.381
Stunting	Albania	0.984	0.178 to 5.459
Stunting	Armenia	0.577	0.278 to 1.199
Stunting	Angola	0.491	0.316 to 0.764
Stunting	Azerbaijan	1.003	0.542 to 1.857
Stunting	Bangladesh	0.895	0.644 to 1.245
Stunting	Burkina Faso	0.580	0.358 to 0.939
Stunting	Benin	1.029	0.796 to 1.332
Stunting	Bolivia	0.763	0.605 to 0.964
Stunting	Burundi	0.480	0.316 to 0.729
Stunting	Congo - Kinshasa	0.762	0.387 to 1.500
Stunting	Congo - Brazzaville	0.499	0.152 to 1.643
Stunting	Côte d’Ivoire	1.022	0.615 to 1.696
Stunting	Dominica	0.215	0.079 to 0.586
Stunting	Egypt	0.967	0.800 to 1.167
Stunting	Ethiopia	0.698	0.392 to 1.244
Stunting	Gabon	0.644	0.363 to 1.146
Stunting	Ghana	0.105	0.012 to 0.906
Stunting	Gambia	0.662	0.419 to 1.048
Stunting	Guinea	0.647	0.418 to 1.001
Stunting	Guatemala	0.719	0.581 to 0.891
Stunting	Guyana	1.042	0.520 to 2.089
Stunting	Honduras	0.692	0.519 to 0.923
Stunting	Haiti	0.469	0.289 to 0.761
Stunting	India	0.889	0.792 to 0.997
Stunting	Jordan	0.642	0.295 to 1.400
Stunting	Kenya	0.732	0.536 to 1.001
Stunting	Comoros	1.204	0.781 to 1.857
Stunting	Kyrgyzstan	0.770	0.482 to 1.228
Stunting	Liberia	0.671	0.213 to 2.118

Outcome	Country	Design-adjusted OR	95% CI
Stunting	Lesotho	0.662	0.303 to 1.446
Stunting	Madagascar	1.081	0.830 to 1.410
Stunting	Mali	0.746	0.562 to 0.990
Stunting	Myanmar (Burma)	0.987	0.609 to 1.598
Stunting	Mauritania	0.537	0.254 to 1.136
Stunting	Maldives	1.069	0.687 to 1.664
Stunting	Malawi	0.875	0.527 to 1.453
Stunting	Mozambique	1.869	0.656 to 5.328
Stunting	Nigeria	0.704	0.578 to 0.857
Stunting	Niger	0.439	0.262 to 0.736
Stunting	Namibia	0.392	0.192 to 0.801
Stunting	Nepal	0.569	0.285 to 1.135
Stunting	Peru	0.568	0.436 to 0.739
Stunting	Papua New Guinea	1.223	0.509 to 2.941
Stunting	Pakistan	0.785	0.396 to 1.558
Stunting	Rwanda	0.755	0.550 to 1.037
Stunting	Sierra Leone	0.937	0.690 to 1.273
Stunting	Senegal	0.977	0.617 to 1.548
Stunting	Eswatini	1.207	0.427 to 3.413
Stunting	Chad	1.225	0.812 to 1.848
Stunting	Togo	1.286	0.737 to 2.245
Stunting	Tajikistan	0.696	0.487 to 0.995
Stunting	Timor-Leste	0.871	0.653 to 1.164
Stunting	Tanzania	0.927	0.597 to 1.437
Stunting	Uganda	0.740	0.527 to 1.039
Stunting	Yemen	0.511	0.409 to 0.639
Stunting	South Africa	0.706	0.355 to 1.405
Stunting	Zambia	2.423	0.931 to 6.304
Stunting	Zimbabwe	0.793	0.562 to 1.121
Underweight	Albania	2.507e+08	3.030e+07 to 2.075e+09
Underweight	Armenia	0.518	0.049 to 5.506
Underweight	Angola	0.715	0.445 to 1.149
Underweight	Azerbaijan	0.387	0.161 to 0.928
Underweight	Bangladesh	0.566	0.395 to 0.810
Underweight	Burkina Faso	1.168	0.723 to 1.887
Underweight	Benin	1.137	0.871 to 1.484
Underweight	Bolivia	0.657	0.427 to 1.012
Underweight	Burundi	0.520	0.299 to 0.904
Underweight	Congo - Kinshasa	0.610	0.226 to 1.651
Underweight	Congo - Brazzaville	0.294	0.076 to 1.129

Outcome	Country	Design-adjusted OR	95% CI
Underweight	Côte d'Ivoire	0.574	0.338 to 0.975
Underweight	Dominica	0.313	0.085 to 1.155
Underweight	Egypt	1.077	0.779 to 1.487
Underweight	Ethiopia	0.625	0.323 to 1.210
Underweight	Gabon	0.956	0.357 to 2.560
Underweight	Ghana	0.145	0.017 to 1.249
Underweight	Gambia	0.631	0.369 to 1.078
Underweight	Guinea	0.508	0.294 to 0.876
Underweight	Guatemala	0.766	0.575 to 1.020
Underweight	Guyana	1.621	0.737 to 3.566
Underweight	Honduras	0.706	0.463 to 1.077
Underweight	Haiti	0.495	0.261 to 0.940
Underweight	India	0.833	0.735 to 0.944
Underweight	Jordan	0.768	0.202 to 2.927
Underweight	Kenya	0.810	0.536 to 1.223
Underweight	Comoros	1.141	0.630 to 2.066
Underweight	Kyrgyzstan	0.516	0.210 to 1.264
Underweight	Liberia	0.095	0.012 to 0.741
Underweight	Lesotho	1.097	0.450 to 2.672
Underweight	Madagascar	0.869	0.634 to 1.191
Underweight	Mali	0.824	0.607 to 1.118
Underweight	Myanmar (Burma)	0.976	0.603 to 1.581
Underweight	Mauritania	0.560	0.209 to 1.502
Underweight	Maldives	0.898	0.497 to 1.623
Underweight	Malawi	0.704	0.347 to 1.428
Underweight	Mozambique	1.913	0.511 to 7.154
Underweight	Nigeria	0.692	0.559 to 0.856
Underweight	Niger	0.439	0.279 to 0.692
Underweight	Namibia	0.239	0.094 to 0.608
Underweight	Nepal	0.365	0.161 to 0.829
Underweight	Peru	0.548	0.359 to 0.836
Underweight	Papua New Guinea	1.095	0.415 to 2.892
Underweight	Pakistan	0.768	0.351 to 1.684
Underweight	Rwanda	0.768	0.402 to 1.467
Underweight	Sierra Leone	1.041	0.720 to 1.504
Underweight	Senegal	0.994	0.587 to 1.683
Underweight	Eswatini	1.796	0.305 to 10.588
Underweight	Chad	1.587	1.070 to 2.356
Underweight	Togo	1.221	0.687 to 2.172
Underweight	Tajikistan	0.267	0.147 to 0.486

Outcome	Country	Design-adjusted OR	95% CI
Underweight	Timor-Leste	0.826	0.643 to 1.062
Underweight	Tanzania	0.603	0.317 to 1.149
Underweight	Uganda	0.880	0.563 to 1.378
Underweight	Yemen	0.640	0.502 to 0.817
Underweight	South Africa	0.466	0.129 to 1.678
Underweight	Zambia	2.797e-08	8.579e-09 to 9.122e-08
Underweight	Zimbabwe	0.617	0.377 to 1.010
OWOB	Albania	1.186	0.524 to 2.683
OWOB	Armenia	0.486	0.236 to 1.001
OWOB	Angola	0.272	0.100 to 0.740
OWOB	Azerbaijan	0.720	0.344 to 1.507
OWOB	Bangladesh	2.733	1.324 to 5.641
OWOB	Burkina Faso	1.383	0.371 to 5.148
OWOB	Benin	1.334	0.668 to 2.661
OWOB	Bolivia	1.090	0.784 to 1.516
OWOB	Burundi	0.690	0.202 to 2.359
OWOB	Congo - Kinshasa	0.443	0.101 to 1.955
OWOB	Congo - Brazzaville	5.625	0.401 to 78.896
OWOB	Côte d’Ivoire	1.211	0.341 to 4.303
OWOB	Dominica	0.241	0.029 to 1.987
OWOB	Egypt	0.978	0.798 to 1.199
OWOB	Ethiopia	1.978	0.719 to 5.444
OWOB	Gabon	2.199	1.054 to 4.589
OWOB	Ghana	1.975	0.154 to 25.329
OWOB	Gambia	0.444	0.101 to 1.961
OWOB	Guinea	0.652	0.260 to 1.637
OWOB	Guatemala	0.877	0.540 to 1.424
OWOB	Guyana	0.398	0.160 to 0.992
OWOB	Honduras	0.942	0.595 to 1.491
OWOB	Haiti	0.549	0.179 to 1.682
OWOB	India	1.484	1.113 to 1.978
OWOB	Jordan	0.968	0.555 to 1.686
OWOB	Kenya	2.623	1.640 to 4.198
OWOB	Comoros	1.011	0.537 to 1.906
OWOB	Kyrgyzstan	0.819	0.529 to 1.267
OWOB	Liberia	1.329e-07	6.429e-08 to 2.749e-07
OWOB	Lesotho	1.237	0.499 to 3.066
OWOB	Madagascar	0.719	0.327 to 1.581
OWOB	Mali	1.346	0.617 to 2.937
OWOB	Myanmar (Burma)	0.026	0.003 to 0.210

Outcome	Country	Design-adjusted OR	95% CI
OWOB	Mauritania	1.661	0.332 to 8.294
OWOB	Maldives	0.703	0.318 to 1.554
OWOB	Malawi	0.772	0.241 to 2.471
OWOB	Mozambique	7.103	1.079 to 46.751
OWOB	Nigeria	0.554	0.293 to 1.048
OWOB	Niger	2.777	1.072 to 7.196
OWOB	Namibia	1.770	0.715 to 4.380
OWOB	Peru	1.338	0.900 to 1.989
OWOB	Papua New Guinea	1.550	0.530 to 4.533
OWOB	Pakistan	0.055	0.007 to 0.446
OWOB	Rwanda	1.396	0.804 to 2.424
OWOB	Sierra Leone	0.645	0.308 to 1.349
OWOB	Senegal	0.432	0.060 to 3.135
OWOB	Eswatini	2.858	0.925 to 8.828
OWOB	Chad	1.460	0.520 to 4.103
OWOB	Togo	2.855	0.857 to 9.512
OWOB	Tajikistan	1.085	0.629 to 1.873
OWOB	Timor-Leste	0.667	0.397 to 1.122
OWOB	Tanzania	0.851	0.256 to 2.829
OWOB	Uganda	0.802	0.422 to 1.524
OWOB	Yemen	0.836	0.483 to 1.448
OWOB	South Africa	1.288	0.673 to 2.462
OWOB	Zambia	1.687	0.276 to 10.313

CI - confidence interval; IPD - individual participant data; OR - odds ratio. Extremely large or small underweight and OWOB estimates reflect sparse-data separation and are shown in scientific notation. These country-specific estimates are supplementary and should not be interpreted as confirmatory evidence.

Table S15. Income-group-specific random-effects pooled odds ratios and heterogeneity statistics from the two-stage individual participant data meta-analysis.

Outcome	Income group	Pooled OR	95% CI	I2, %	tau2	Q	P heterogeneity	Studies, n
Anaemia	Upper-middle-income	0.907	0.772 to 1.065	24.3	0.020	11.889	0.220	10
Anaemia	Lower-middle-income	0.968	0.906 to 1.034	24.5	2.737e-06	29.125	0.141	23
Anaemia	Low-income	0.995	0.895 to 1.106	0.0	0.000	10.175	0.809	16
Stunting	Upper-middle-income	0.686	0.562 to 0.836	40.6	0.034	16.835	0.078	11
Stunting	Lower-middle-income	0.807	0.745 to 0.874	27.6	0.010	38.665	0.086	29
Stunting	Low-income	0.782	0.662 to 0.925	65.2	0.072	48.820	<0.001	18
Underweight	Upper-middle-income	3.390	0.103 to 111.184	97.1	34.483	347.567	<0.001	11
Underweight	Lower-middle-income	0.728	0.642 to 0.826	45.6	0.045	51.431	0.004	29
Underweight	Low-income	0.289	0.045 to 1.860	98.0	16.079	842.823	<0.001	18
OWOB	Upper-middle-income	0.960	0.706 to 1.305	50.1	0.124	20.028	0.029	11
OWOB	Lower-middle-income	1.005	0.802 to 1.260	63.8	0.177	71.831	<0.001	27
OWOB	Low-income	0.487	0.081 to 2.928	99.0	14.752	1713.962	<0.001	18

CI - confidence interval; I2 - percentage heterogeneity; IPD - individual participant data; OR - odds ratio; Q - Cochran's Q; tau2 - estimated between-study variance. Pooled estimates were considered supplementary. Underweight and OWOB estimates in some income groups were particularly unstable and should not be interpreted as confirmatory evidence.

Table S16. Effect modification of minimum dietary diversity-health outcome associations by wealth, residence, and geographic region.

Outcome	Moderator	Level	aOR per 1-point MDD	95% CI	P	P interaction
OWOB	Wealth quintile	Quintile 1 (poorest)	0.985	0.942 to 1.029	0.495	0.451
OWOB	Wealth quintile	Quintile 2	0.980	0.938 to 1.024	0.371	0.451
OWOB	Wealth quintile	Quintile 3	1.021	0.972 to 1.072	0.416	0.451
OWOB	Wealth quintile	Quintile 4	1.024	0.973 to 1.078	0.357	0.451
OWOB	Wealth quintile	Quintile 5 (richest)	1.026	0.973 to 1.081	0.346	0.451
OWOB	Residence	Urban	1.035	0.998 to 1.075	0.066	0.206
OWOB	Residence	Rural	1.006	0.978 to 1.034	0.701	0.206
OWOB	Geography	Africa	1.036	1.003 to 1.070	0.034	0.720
OWOB	Geography	Asia	1.011	0.971 to 1.053	0.601	0.720
OWOB	Geography	Europe	1.044	0.846 to 1.289	0.687	0.720
OWOB	Geography	Latin America & Caribbean	1.006	0.954 to 1.060	0.837	0.720
OWOB	Geography	Oceania	0.890	0.656 to 1.208	0.454	0.720
Stunting	Wealth quintile	Quintile 1 (poorest)	1.049	1.027 to 1.071	<0.001	<0.001
Stunting	Wealth quintile	Quintile 2	0.997	0.975 to 1.020	0.809	<0.001
Stunting	Wealth quintile	Quintile 3	0.992	0.967 to 1.017	0.506	<0.001
Stunting	Wealth quintile	Quintile 4	0.984	0.958 to 1.012	0.263	<0.001
Stunting	Wealth quintile	Quintile 5 (richest)	0.975	0.942 to 1.008	0.132	<0.001
Stunting	Residence	Urban	0.948	0.926 to 0.971	<0.001	<0.001
Stunting	Residence	Rural	1.000	0.986 to 1.013	0.944	<0.001
Stunting	Geography	Africa	0.974	0.958 to 0.991	0.002	<0.001
Stunting	Geography	Asia	0.989	0.970 to 1.008	0.261	<0.001
Stunting	Geography	Europe	1.195	0.630 to 2.269	0.585	<0.001
Stunting	Geography	Latin America & Caribbean	0.915	0.886 to 0.945	<0.001	<0.001
Stunting	Geography	Oceania	1.098	0.901 to 1.338	0.353	<0.001
Underweight	Wealth quintile	Quintile 1 (poorest)	0.990	0.966 to 1.015	0.427	0.340
Underweight	Wealth quintile	Quintile 2	0.985	0.959 to 1.013	0.302	0.340
Underweight	Wealth quintile	Quintile 3	0.957	0.929 to 0.987	0.004	0.340

Outcome	Moderator	Level	aOR per 1-point MDD	95% CI	P	P interaction
Underweight	Wealth quintile	Quintile 4	0.966	0.935 to 0.998	0.035	0.340
Underweight	Wealth quintile	Quintile 5 (richest)	0.960	0.924 to 0.998	0.040	0.340
Underweight	Residence	Urban	0.938	0.911 to 0.966	<0.001	0.169
Underweight	Residence	Rural	0.960	0.945 to 0.975	<0.001	0.169
Underweight	Geography	Africa	0.943	0.924 to 0.964	<0.001	<0.001
Underweight	Geography	Asia	0.947	0.928 to 0.966	<0.001	<0.001
Underweight	Geography	Europe	1.800	1.487 to 2.181	<0.001	<0.001
Underweight	Geography	Latin America & Caribbean	0.915	0.873 to 0.959	<0.001	<0.001
Underweight	Geography	Oceania	0.940	0.749 to 1.179	0.590	<0.001
Anaemia	Wealth quintile	Quintile 1 (poorest)	1.008	0.983 to 1.033	0.545	0.213
Anaemia	Wealth quintile	Quintile 2	1.002	0.977 to 1.027	0.889	0.213
Anaemia	Wealth quintile	Quintile 3	1.039	1.011 to 1.068	0.006	0.213
Anaemia	Wealth quintile	Quintile 4	1.032	1.002 to 1.063	0.040	0.213
Anaemia	Wealth quintile	Quintile 5 (richest)	1.006	0.975 to 1.037	0.719	0.213
Anaemia	Residence	Urban	1.021	0.999 to 1.044	0.066	0.153
Anaemia	Residence	Rural	1.002	0.986 to 1.017	0.846	0.153
Anaemia	Geography	Africa	0.985	0.965 to 1.005	0.145	0.027
Anaemia	Geography	Asia	1.026	1.005 to 1.047	0.014	0.027
Anaemia	Geography	Europe	0.922	0.770 to 1.105	0.379	0.027
Anaemia	Geography	Latin America & Caribbean	0.987	0.955 to 1.020	0.427	0.027

aOR - adjusted odds ratio per 1-point increase; CI - confidence interval; MDD - minimum dietary diversity; OWOB - overweight/obesity. Interaction terms were entered one at a time in the survey-weighted models.

Checklist S1. STROBE Statement checklist of items that should be included in reports of cross-sectional studies.

Section	Item No	Recommendation	Page / location where reported
	1	(a) Indicate the study's design with a commonly used term in the title or the abstract	Main manuscript: Title and Abstract.
	1	(b) Provide in the abstract an informative and balanced summary of what was done and what was found	Main manuscript: Abstract.
Introduction	2	Explain the scientific background and rationale for the investigation being reported	Main manuscript: INTRODUCTION, background and rationale.
Introduction	3	State specific objectives, including any prespecified hypotheses	Main manuscript: INTRODUCTION, final paragraph stating the study aims.
Methods	4	Present key elements of study design early in the paper	Main manuscript: METHODS, Data source and study population.
Methods	5	Describe the setting, locations, and relevant dates, including periods of recruitment, exposure, follow-up, and data collection	Main manuscript: METHODS, Data source and study population; Supplement, Text S1.
Methods	6	(a) Give the eligibility criteria, and the sources and methods of selection of participants	Main manuscript: METHODS, Data source and study population; Supplement, Tables S1-S3.
Methods	7	Clearly define all outcomes, exposures, predictors, potential confounders, and effect modifiers. Give diagnostic criteria, if applicable	Main manuscript: METHODS, Study variables and Statistical analyses.
Methods	8*	For each variable of interest, give sources of data and details of methods of assessment (measurement). Describe comparability of assessment methods if there is more than one group	Main manuscript: METHODS, Study variables; Supplement, Text S1.
Methods	9	Describe any efforts to address potential sources of bias	Main manuscript: METHODS, Statistical analyses and DISCUSSION, limitations; Supplement, Table S3.
Methods	10	Explain how the study size was arrived at	Main manuscript: METHODS, Data source and study population.
Methods	11	Explain how quantitative variables were handled in the analyses. If applicable, describe which groupings were chosen and why	Main manuscript: METHODS, Minimum dietary diversity definition and Statistical analyses; Supplement, Text S1.
Methods	12	(a) Describe all statistical methods, including those used to control for confounding	Main manuscript: METHODS, Statistical analyses; Supplement, Text S1.
Methods	12	(b) Describe any methods used to examine subgroups and interactions	Main manuscript: METHODS, Statistical analyses; Supplement, Table S16.
Methods	12	(c) Explain how missing data were addressed	Main manuscript: METHODS, Statistical analyses; Supplement, Tables S2-S3.
Methods	12	(d) If applicable, describe analytical methods taking account of sampling strategy	Main manuscript: METHODS, Statistical analyses; Supplement, Text S1.
Methods	12	(e) Describe any sensitivity analyses	Main manuscript: METHODS, Statistical analyses; Supplement, Tables S7-S16.
Results	13*	(a) Report numbers of individuals at each stage of study—eg numbers potentially eligible, examined for eligibility, confirmed eligible, included in the study, completing follow-up, and analysed	Main manuscript: METHODS, Data source and study population; RESULTS, Sample characteristics.

Section	Item No	Recommendation	Page / location where reported
Results	13*	(b) Give reasons for non-participation at each stage	Main manuscript: METHODS, Data source and study population; Supplement, Tables S1-S3.
Results	13*	(c) Consider use of a flow diagram	Not applicable: secondary analysis of existing survey data; no recruitment flow diagram was used.
Results	14*	(a) Give characteristics of study participants (eg demographic, clinical, social) and information on exposures and potential confounders	Main manuscript: Table 1 and RESULTS, Sample characteristics.
Results	14*	(b) Indicate number of participants with missing data for each variable of interest	Main manuscript: METHODS, Data source and study population; Supplement, Tables S2-S3.
Results	15*	Report numbers of outcome events or summary measures	Main manuscript: RESULTS, Cross-country distribution of malnutrition; Supplement, Table S6.
Results	16	(a) Give unadjusted estimates and, if applicable, confounder-adjusted estimates and their precision (eg, 95% confidence interval). Make clear which confounders were adjusted for and why they were included	Main manuscript: RESULTS, association analyses; Supplement, Tables S7-S16 and Figures S1-S2.
Results	16	(b) Report category boundaries when continuous variables were categorized	Main manuscript: METHODS, Minimum dietary diversity definition and Statistical analyses; Supplement, Text S1.
Results	16	(c) If relevant, consider translating estimates of relative risk into absolute risk for a meaningful time period	Not applicable: cross-sectional odds ratios were not converted to absolute risks over time.
Results	17	Report other analyses done—eg analyses of subgroups and interactions, and sensitivity analyses	Main manuscript: RESULTS, sensitivity and effect-modification analyses; Supplement, Tables S7-S16 and Figures S1-S2.
Discussion	18	Summarise key results with reference to study objectives	Main manuscript: DISCUSSION, opening summary.
Discussion	19	Discuss limitations of the study, taking into account sources of potential bias or imprecision. Discuss both direction and magnitude of any potential bias	Main manuscript: DISCUSSION, strengths and limitations.
Discussion	20	Give a cautious overall interpretation of results considering objectives, limitations, multiplicity of analyses, results from similar studies, and other relevant evidence	Main manuscript: DISCUSSION and CONCLUSIONS.
Discussion	21	Discuss the generalisability (external validity) of the study results	Main manuscript: DISCUSSION, strengths and limitations and policy implications.
Other information	22	Give the source of funding and the role of the funders for the present study and, if applicable, for the original study on which the present article is based	Main manuscript: Funding statement and Authorship contributions.

STROBE - Strengthening the Reporting of Observational Studies in Epidemiology. The recommendation wording follows the official cross-sectional checklist available from https://www.strobe-statement.org/checklists/. Section-based locations are used to remain reliable if production pagination changes.
